# The Metabolic Matrix: Re-engineering ultraprocessed foods to feed the gut, protect the liver, and support the brain

**DOI:** 10.3389/fnut.2023.1098453

**Published:** 2023-03-30

**Authors:** Timothy S. Harlan, Rachel V. Gow, Andreas Kornstädt, P. Wolfram Alderson, Robert H. Lustig

**Affiliations:** ^1^Division of General Internal Medicine, George Washington University School of Medicine and Health Sciences, Washington, DC, United States; ^2^Institute of Psychiatry, Psychology and Neuroscience (IoPPN), King’s College London, London, United Kingdom; ^3^Perfact, San Francisco, CA, United States; ^4^Human & Environmental Health Department, Kuwaiti Danish Dairy Company, Kuwait City, Kuwait; ^5^Department of Pediatrics and Institute for Health Policy Studies, University of California, San Francisco, San Francisco, CA, United States

**Keywords:** metabolic health, brain health, ultraprocessed food, nutrition, re-engineering

## Abstract

Ultraprocessed food is established as a metabolic disruptor acting to increase adiposity, reduce mitochondrial efficiency, drive insulin resistance, alter growth, and contribute to human morbidity and mortality. Consumer packaged goods (CPG) companies are beginning to understand the detrimental impact of the food they market, and have employed substitution strategies to reduce salt, sugar, and fat. However, the harms of ultraprocessed foods are far more complex than any single component, and are not ameliorated by such simple substitutions. Over the past 2 years, the authors have worked with the Kuwaiti Danish Dairy Company (KDD) to conduct a comprehensive scientific evaluation of their entire commercial food and beverage portfolio. Assay of the macronutrients, micronutrients, additives, and toxins contained in each of their products was undertaken to determine the precise nature of each product’s ingredients as well as the health impacts of processing. The authors formed a Scientific Advisory Team (SAT) and developed a tiered “Metabolic Matrix” founded in three science-based principles: (1) *protect the liver*, (2) *feed the gut*, and (3) *support the brain*. The Metabolic Matrix categorizes each product and provides the criteria, metrics, and recommendations for improvement or reformulation. Real-time consultation with the KDD Executive and Operations teams was vital to see these procedures through to fruition. This scientific exercise has enabled KDD to lay the groundwork for improving the health, well-being, and sustainability of their entire product line, while maintaining flavor, economic, and fiscal viability. This process is easily transferrable, and we are sharing this effort and its approaches as a *proof-of-concept*. The key aim of our work is to not only make ultraprocessed food *healthier* but to urge other food companies to implement similar analysis and reformulation of their product lines to improve the metabolic health and well-being of consumers worldwide.

## 1. Introduction

Chronic metabolic diseases (including type 2 diabetes, hypertension, dyslipidemia, cardiovascular disease, cancer, dementia, polycystic ovarian disease, and non-alcoholic fatty liver disease) are now rampant throughout both the developed and developing world. These diseases are increasing in prevalence, severity, and as a percentage of total healthcare costs worldwide (1). Each of these chronic metabolic diseases are associated with dysfunctional mitochondrial energetics, resulting in the phenomenon of insulin resistance, which foments other altered cellular processes resulting in chronic disease. The key questions are: (1) where does this insulin resistance come from; and (2) why has it worsened over the last 50 years?

A standard misconception among health professionals is that chronic disease is the inevitable result of the aging process. This does not explain why children as young as the first decade now exhibit these same biochemical processes, and many children now manifest two diseases that were rarely seen in this age group: type 2 diabetes and fatty liver disease. In fact, many neonates harbor increased adiposity, a result of altered foetal energy partitioning (2–4).

A second misconception is that the rise in prevalence and severity of obesity is self-determined due to an increased prevalence of the vices of gluttony and sloth. This belief is countered based on the physiology of three phenomena which document global involuntary perturbations in cellular biochemistry. First, laboratory animals in captivity are experiencing an increase in weight; inferring a global metabolic insult not restricted to humans (5). Second, body temperature has declined over the past 150 years in the United States commensurate with the rise in obesity; inferring a subcellular defect in mitochondrial beta-oxidation and heat generation. Third, all vertebrate life on this planet is exposed to environmental obesogens, many of which are found in the commercial food supply, with some directly affecting adipose tissue differentiation, and others impacting mitochondrial beta-oxidation, and driving weight gain exclusive of calories (6).

A third misconception is that obesity and chronic disease are the same phenomenon. Rather, it must be pointed out that 20% of individuals who are obese are metabolically healthy (7) with normal lifespan and health span, and expected biochemical markers of aging, such as normal length telomeres (8). Conversely. 40% of individuals of normal weight manifest one or more chronic metabolic diseases. In the United States, up to 93% of the adult population manifest some aspect of metabolic dysfunction (9), while only 65% of individuals are either overweight or obese (10). People of normal weight also develop these diseases, which are increasing in prevalence in countries with low obesity rates as well. Therefore, there must be a more global, and likely more obscure, exposure that explains the high prevalence of insulin resistance and chronic disease in populations with low obesity rates.

A fourth misconception is that most clinicians mistakenly attribute the growing rise of non-communicable diseases (NCDs) to fat depots which are outwardly noticeable. This is also untrue, based on two endocrinopathies that highlight the dichotomy between obesity and chronic disease. First, the “Little Women of Loja” is a founder-effect cohort in Ecuador who are growth hormone-receptor deficient and who become markedly obese yet are protected from chronic metabolic disease such as diabetes and heart disease (11). Conversely, patients with lipodystrophy are devoid of subcutaneous fat, but instead develop liver and muscle (ectopic) fat and severe insulin resistance (12). It is not the fat you can see that causes disease; it is the fat you cannot see — and many people of normal weight harbor ectopic fat and insulin resistance.

The fifth and final misconception is that the cause of chronic disease is the quantity of the food consumed according to the metric of “calories.” Rather, it is the quality of the food consumed that contributes to insulin resistance. The Standard American Diet (SAD; also known as the Western Diet or the Processed Food Diet), replete with ultraprocessed foods, acts as endocrine disruptors that drive adiposity and alter mitochondrial ATP production. The recent advent, validation, and utilization of the NOVA classification of food processing (13) demonstrates that Group 4, i.e., the ultraprocessed food category, portends the greatest risks of morbidity and mortality, as numerous culturally diverse studies illustrate that ultraprocessed food consumption is correlated with obesity (14), diabetes (15), heart disease (16), cancer (17), dementia (18), and other mental health disorders (19). In short, obesity and chronic disease are not the same in the same way that different sources of calories are not the same (20).

Although many ingredients in ultraprocessed food are associated with metabolic derangement (21), perhaps the most studied and consistently vilified by both public health experts (22) and commercial interests (23–26) is sugar. It is also the most malleable, as the food industry develops numerous non-nutritive sweetener alternatives to replace sugar in its recipes. Indeed, many consumer packaged goods (CPG) companies have made initial efforts to reduce the sugar content of their portfolios to improve the quality of their ultraprocessed products. Also, a group of start-ups have formed a nascent Alliance to Combat Excessive Sugar (ACES) (27).

However, ultraprocessed foods are detrimental to human health across several parameters, including macronutrient and micronutrient composition, fiber, effects of food additives, toxins, heat exposure, and packaging. Recently, academic investigators have provided a framework for the reformulation of processed foods to improve health and sustainability (28). We believe that to make ultraprocessed food healthier, a more scientific approach that considers the various metabolic effects of food ingredients and processing is required. Instead of “*Can we make healthy food tasty?*,” we asked “*Can we make tasty food healthy?*”

Over the years 2020–2022, we have worked with the Executive Team at KDD to research and reimagine their entire 180-item portfolio to develop best-in-class (nourishing, delicious, affordable, commercially viable) food and beverages that support metabolic health and well-being. The key focus of this re-engineering effort was to identify: the composition of the food (ingredients), what is done to the food (processing), and the metabolic impact of the food (metabolism). While we were mindful of cost of both ingredients and procedures throughout, the analysis was not cost-driven; rather we strived to make recommendations to improve KDD’s metabolic health portfolio and leave sales and marketing to the KDD leadership. Our results below are offered as a proof-of-concept and as a roadmap for other companies who wish to engage in a similar exercise, for the benefit of both their corporation and their consumers.

## 2. Materials and methods

Multiple systems have been constructed in an effort at evaluation of processed foods for their impact on health. The modern era may be considered to have begun with the Food and Drugs Act of 1906, and in the ensuing decades revisions to the law expanded labeling to include food standards and guidance regarding both safe handling and ingredients. Over the last four decades efforts at consumer education, especially at the point of sale, have brought on simple systems such as the Nutrition Facts labeling system mandated by the Food and Drug Administration (FDA) in 1990. However, a wide variety of point-of-sale alerts for consumers have not shown efficacy with respect to improvement in dietary habits.

While evidence exists that these systems enhance consumer knowledge, there is little convincing evidence of significant impact on health outcomes (29–31). The challenge of utilizing such systems is the reliance on basic nutrition information, primarily macronutrients and micronutrients, which fail to account for the totality of a particular food. This is especially true for consumer packaged goods (CPG) and ultraprocessed foods which carry potential health impact beyond the amount of fat, carbohydrates, protein, or sodium.

Non-governmental food labeling systems have been developed with mixed success, and many have come under scrutiny for undue influence from multinational CPG manufacturers (32). As with other front-of-pack label schemes, private labeling programs have relied primarily on macronutrient and micronutrient content (33). More recent systems have sought to evaluate the impact of processed foods (34). We sought to craft a prospective system that helps CPG companies with the formulation of their products and create a tiered Metabolic Matrix that allows development of foods that are healthy from the very start of product development. Below are the five components developed to integrate metabolic health, food science, and industrial engineering, and blend them into a cohesive strategy exportable to other venues.

### 2.1. The Metabolic Matrix

We evaluated the criteria for potential ingredients based on their metabolic impact rather than on their nutritional content. The criteria of how ingredients in a particular product might contribute to an adverse health impact focused on detriments to gut, liver, and brain function. While all organ systems are involved in metabolic health, these three have the most significant impact on overall metabolism and disease, because dysfunctions of any of these three can lead to resultant dysfunction in other organs, whereas the converse is not necessarily true. For instance: (a) hepatic insulin resistance can lead to pancreatic insulin oversecretion, but the pancreatic insulin oversecretion does not lead to hepatic insulin resistance (35); (b) gut inflammation can lead to insulin resistance, but insulin resistance does not lead to gut inflammation (36); or (c) brain insulin resistance leads to obesity, but obesity does not necessarily lead to brain insulin resistance (37). The overarching features of each pillar of the Metabolic Matrix are detailed in Table 1.

**Table 1:** Rationale and strategies of the Metabolic Matrix to improve health.

Feed the gut	1. Soluble fiber
	2. Insoluble fiber
	3. Reduce processed carbohydrates
	4. Whole intact food (cellular) matrix
	5. Provide prebiotic nourishment (dietary fiber)
	6. Replace probiotic nourishment (gut microbiota)
Protect the liver	1. Fructose reduction
	2. Reduce total sugar intake
	3. Appropriate hydration
	4. Reduce environmental toxins
	5. Reduce glycemic load
Support the brain	1. Through nutrient-dense foods
	2. With healthy and brain-essential fats
	3. With healthy proteins providing sufficient and appropriate amino acids
	4. With “brain-selective” nutrients which help govern neurotransmitter function

The first pillar is to “*feed the gut*.” The neutral activity of fiber and its lack of absorption and metabolism from the gut indicates that the health benefits are focused in the intestine but offer significant downstream impacts. There is clear evidence that consumption of higher quality fiber offers a significant reduction in morbidity and mortality (38–40). Foods that provide quality fiber (both soluble and insoluble) provide a significant impact on glycemic control, beginning in the gut (40). Positive impacts of fiber on the gut microbiota (38, 39) are well documented and offer opportunity for improvement in the health of the gut microbiota. Colonic fermentation of fibers by gut bacteria have been shown to produce short-chain fatty acids (SFCA) as metabolic byproducts, which provide nourishment for colonic bacteria and serve as an anti-inflammatory agent (41). Health benefits such as improved cardiovascular risk appear to be mediated by the downstream impact of SFCA on the integrity of the gut barrier as well as improved glucose and lipid metabolism (42–44).

The second pillar is to “*protect the liver*.” While the liver is the primary detoxification organ for most poisons, it is abundantly clear that the liver has limits to its capacity, i.e., “the dose determines the poison.” Toxins in food can overwhelm the cytochrome P450 system (e.g., cadmium, glyphosate), or the mitochondrial TCA cycle (e.g., ethanol, *trans*-fats, branched-chain amino acids, fructose). In either case, the liver becomes dysfunctional, substrates may be diverted into the production of liver fat (45), with ensuing hepatic insulin resistance resulting in hyperinsulinemia, which drives aberrant cellular growth and foments chronic metabolic disease (46). Unfortunately, these compounds are highly abundant in ultraprocessed food (47), and there are no medications that can protect cytochromes or mitochondria. Reduction in substrate exposure is the only rational preventative measure.

The third pillar is to “*support the brain*.” The Western diet is characterized as the habitual consumption of ultraprocessed food products, characterized by an elevated intake of omega-6 fatty acids (which in excess can promote inflammation) (48), with simultaneous insufficiency in brain-essential omega-3 fats, excessive sugar and sodium, reduced micronutrient intake, and a high intake of refined carbohydrates (49). The omega-6 to omega-3 ratio consistent with Western-type dietary patterns appears to be a primary contributing factor to the premature development of all NCDs, including metabolic dysfunction such as heart disease and stroke (50, 51), neurodegenerative diseases (52); addiction (53, 54), and depression (55–57).

The correlation between *diseases of the body* and *diseases of the mind* is well established (58). There are a plethora of research articles reporting this bi-directional relationship, in particular highlighting depression as a key predictor of cardiovascular disease with estimates of up to 47% of patients with coronary heart disease experiencing major depression (59–61).

Scientific advances have enabled us to better understand that inflammation and endocrine pathways interact and are implicated in brain health at molecular and cellular levels. Indeed, inflammation is now recognized as a key driver of disorders of mental health. Processed food consumption is marked by (a) elevated intake of omega-6 fatty acids *via* the inclusion of omega-6-rich, industrially produced, cheap, refined, often hydrogenated vegetable oils (e.g., soybean oil) which in excess can contribute to hyperinsulinemia and the development of insulin resistance (62), and (b) insufficient intakes of anti-inflammatory, long-chain, polyunsaturated omega-3 dietary essential fatty acids, specifically docosahexaenoic acid (DHA) and eicosapentaenoic acid (EPA). Coupled with an elevated intake of fructose, these both contribute to metabolic disease by promoting brain insulin resistance and increasing risk of cognitive dysfunction (63).

While the three pillars of the Metabolic Matrix focus on ingredient selection during development of products, CPG companies now have a clear mandate for societal impact including the importance of protecting their consumers and acting responsibly in the wider scope of global nutrition (64). These missions must be balanced by the commercial imperative of owners and shareholders with concerns of environmental sustainability and employee well-being.

These three pillars of Feed, Protect, and Support can be applied across a company’s nutritional, societal, and commercial efforts in a way that benefits consumers, employees, and shareholders as noted in Table 2.

**Table 2:** Benefits of the Metabolic Matrix from a societal and commercial standpoint.

**Key aims**	**Societal**	**Commercial**
**Feed**	**Feed** the world (in an optimized and sustainable fashion)	**Feed** employee well-being through positive business practices and communications
**Protect**	**Protect** the customer (community)	**Protect** brand integrity through corporate responsibility and sustainable practices
**Support**	**Support** global nutrition education	**Support** long-term financial growth through innovative product development and market strategies

### 2.2. Development of the TIERS classification

There are many shortcomings of the multiple systems that have been compiled with an effort at evaluating processed foods and their impact on health: (a) a focus on macronutrients and micronutrients (instead of on health outcomes); (b) the limited impact on changing consumer behavior; (c) a retrospective point of view that pays little attention to guiding manufacturers to create healthy products; and (d) a propensity to be corrupted by political influence (65). In addition, these systems expose structural issues which are no less severe.

#### 2.2.1. Oversimplification

Previous systems like the UK FSA’s “traffic light” system, which almost doubled the permissible amount of sugar only 6 years after its inception in 2006 under industry pressure, employ reference amounts of 100 g across all food groups. The FDA is only now addressing the criticisms dealing with unrealistic serving sizes (66). While these reports address serving size manipulation concerns, it creates issues for manufacturers of butter, olive oil, salt, or sugar whose products cannot escape being allocated a red traffic light label even though oil, salt, and sugar are seldom consumed in 100-gram portions (67). Knowing that traffic lights are better ignored for some product groups undermines the trust and thus the impact of such oversimplified systems. Similarly, front-of-package warnings, such as those employed in Mexico (68), have not been successful and are often ignored by consumers.

#### 2.2.2. Blind spots

Introducing categories like the French NutriScore system can help to address oversimplification issues by employing specific criteria for different food categories (33). However, this may lead to the wholesale exclusion of relevant food groups. For example, the Nordic Keyhole system explicitly excludes most drinks and sweets as ineligible, even though naturally flavored water or unsweetened chocolate are arguably little cause for concern (69).

#### 2.2.3. Averaging scores

To deal with the complexity of factors, systems like NutriScore or Food Compass (70) use a mathematical scoring system that awards positive points for attributes deemed healthful and negative points for those deemed detrimental. Points are then added up to obtain a final score. While this might appear on paper to be a solid idea, this “poison A + antidote B = neutral” approach is another example of oversimplification because the simple addition and fortification of vitamins and minerals cannot offset the presence of harmful ingredients. Employing a system that averages scores makes it easy to gloss over negative aspects to further an unduly positive view of products. These shortcomings prevented our team from relying on existing systems, so we developed a system that aimed to address and overcome some of these challenges.

#### 2.2.4. Applying a finer-grained approach when it comes to nutrients

As outlined previously, evaluating a food item’s merits based on just the information that appears on food labels, such as “unsaturated fatty acids” or “sugars” is insufficient given the different qualities of members of each of these groups. For instance, the omega-3’s alpha-linolenic acid (ALA), DHA, and EPA play different functional and structural roles in bodily cells and thus need to be considered individually. The same holds true for the sugars: glucose, fructose, and galactose, which are metabolized differently (71) and cannot reasonably be treated equally.

#### 2.2.5. Moving beyond macronutrients and micronutrients and considers ingredients as well

While the Nutrition Facts label covers many important aspects, it is not sufficient to get a full picture of a product’s qualities. For example, many additives have nondescript nutrient profiles and cannot be dealt with from a viewpoint that is constrained by a “nutrients only” perspective. Expanding the scope from “nutrients only” to ingredients enables us to take a refined stance on additives according to current research on their potential harmfulness. It also allows us to precisely determine ultraprocessing levels rather than trying to derive them from nutrient profiles (72).

### 2.3. Food additives review

There are ~3,975 food additives cited in the FDA “Substances Added to Food” list (73). We evaluated and reviewed two hundred and fifty-eight food additives in the first phase (see Supplementary Tables 1–5 containing additives with potential harms). Literature reviews were undertaken in relation to each category of additives and summary reports were compiled. Substances were evaluated first and foremost for metabolic impact, considering the available research, cautions, concerns, and limits suggested by regulatory bodies. The broad criteria of “no harmful additives” was arrived at with the intent of establishing a general paradigm of applying the precautionary principle and a mission to investigate, monitor, refine, reduce, or replace ingredients that may have negative metabolic impacts. This exercise aimed to establish a systematic and ongoing approach that can be updated given that new substances are being added to the food supply on a regular basis.

While considering conventional thresholds or ranges for food additives is important, limiting decision-making to per-serving limits established by regulatory bodies discounts the potential of environmental exposures. Human exposure to a given substance can vary widely depending on age, health conditions (both communicable and NCD), food culture, dietary preferences, consumption of ultra-processed foods, and more. Additionally, little is known about how additives are chemically altered by heat, pressure, processing, transportation, storage, and food preparation. It is important to note that many of these additives are combined in complex formulations, and little research is available on their interactions with other substances. Food and beverage companies committed to consumer health and safety should strive to exceed current regulatory standards.

#### 2.3.1. Product categorization without excluding any categories

By avoiding the shortcomings of oversimplification and blind spots, we can be very specific about rule-scoping. For example, it is possible to be lenient on emulsifiers where they have an appropriate place, but to be stricter when they are unnecessary, such as fermented milk products prepared from fresh ingredients.

#### 2.3.2. Meaningful serving sizes as references

While we see the merits of using 100 g as a reference quantity across the board to negate political influence, we have argued that the negative consequences of this oversimplification do more harm than specific serving size adjustments for evaluation purposes. For example, we feel that 100 g are a useful quantity for nutrition labels, but serving sizes are more relevant for evaluating a food’s impact.

#### 2.3.3. Tiering system

Since “poison A + antidote B = neutral” carries little weight, we bundled these criteria into Tiers (see Supplementary Tables 6–9). As these criteria are not equal, they cannot cancel each other out by means of score averaging. Instead, we grouped Tiers criteria in a layered way that places harmful criteria at the bottom (Tier III: harm reduction), basic remediation in the middle (Tier II: compensating deficiencies), and other desirable criteria at the top (Tier I: additional benefits). That means that even a product containing cold-pressed olive oil and added vitamin B_6_ and B_12_ will still be considered a Tier III product if it has more than 5 g of omega-6 fatty acids per serving. Likewise, a product cannot progress to Tier I unless it addresses all relevant Tier II deficiencies (e.g., at least 400 IU Vitamin D per serving). In a second step that goes beyond fixing structural issues of existing systems, this analysis adds qualities to our knowledge not found in combination with current extant systems.

While it is relevant to look at the “soil to mouth” properties of food, it is the food’s impact after it has entered the cell that eventually determines how to evaluate its metabolic merits. The Tiers system may be tailored to address the needs of a particular population. Even though we think that Tiers III and I can be applied widely, Tier II has been developed with a focus on deficiencies in the Middle East and North African (MENA) region that is of particular interest to KDD. It can therefore be more specific than systems that aim at universal applicability. With additional research, Tier II (and III and I if required) can be adapted to other populations.

### 2.4. Organizational structure

The Science Advisory Team (SAT) was commissioned by the leadership of KDD but had no role in its constitution other than selecting Dr. Robert Lustig of UCSF as the lead (unpaid) chairperson. The team members were then chosen by Dr. Lustig to bring a diverse set of scientific qualifications and expertise to the team. Actual or even perceived bias undermines stakeholder trust and thus a system’s acceptance (74). Therefore, our goal was to be unbiased, and neither KDD, other corporations, industry associations, nor governmental agencies had any influence. Our system’s criteria are evidence-based and will be revised accordingly to reflect new findings. SAT members were paid as consultants by KDD but reported directly to Dr. Lustig. The SAT maintained complete independence and autonomy in expressing scientific opinions and deciding on the deliverables, but interacted with key KDD department heads using a cross-team approach, including product development, production, information technology, marketing, sales, and executive leadership. The SAT was given complete access to all product and ingredient information, including nutrition facts, specifications for ingredients, recipes, production practices, food regulations, marketing, sales, and business strategy. Feedback was exchanged, and then a final highly detailed report was provided by the SAT, covering all ingredients in KDD’s portfolio, sorting each of KDD’s 180 products into tiers and allowing all existing and future product developments to have clear pathways toward achieving maximum metabolic impact.

### 2.5. The TIERS document

The TIERS framework is the core tool for implementing the Metabolic Matrix, i.e., *feed the gut, protect the liver, and support the brain*. The SAT developed the TIERS as a multi-level dynamic and progressive framework based upon evidence-based criteria and filters that are used to develop products to achieve optimal metabolic health status, in addition to other criteria such as transparency (e.g., presence of allergens), structure–function claims, animal welfare and environmental concerns, traceable sourcing, independent testing, and establishing minimum thresholds for key dietary vitamins, minerals, fiber, and fats.

To assign a TIERS status, comprehensive product data must be collected, including ingredients and their components, independent testing focused on a wide range of criteria, product data, packaging, ingredient specifications, nutrition facts, and sales and marketing data. The comprehensive body of data is collated and converted into a standardized data format (JSON) and input into the *Perfact* food data science platform. Highly detailed reports were generated assigning TIERS status in addition to providing recommendations for evolving the products to deliver optimal metabolic impacts in addition to other qualities inspired by sustainable development goals and other international standards established for improving human and environmental health.

Product TIERS include TIER III (conventional foods and beverages), TIER II C (focused on healthy fats, sugars, additives, salt, heavy metals, pesticides, and antibiotics), TIER II B (adding vegetable oil criteria), TIER II A (adding minimum targets for vitamins, minerals, fatty acids, and fiber), TIER I B (adding additional targets for vitamins, allergen informatics, health benefits backed by science, independent testing, cold-pressed oils, and amino acids), and TIER I A (adding criteria for environmental sustainability, traceable sourcing). Once classified in TIERS, a given product may require a change in formulation to ascend to the next level. If reformulation is not possible, then it may be discontinued and a new product developed with the intention of replacing it.

#### 2.5.1. Assays of each product (Eurofins)

With a commission from the KDD Executive Leadership, the SAT developed multiple tools for re-imagining the product portfolio based upon a detailed qualitative and quantitative framework. The tools include the Metabolic Matrix, the Progressive Product Tiers, an advanced data framework, and a compendium of scientific reports, functional narratives, research, strategies, and tactics.

The initiative involved assembling a multi-dimensional database framework evaluating all KDD products, comprising over 75,000 data points from multiple sources, including ingredient/component specification sheets, EHSA Research Genesis R&D suite of product development and labeling software, laboratory analysis of products and ingredients, marketing and sales data, packaging, and sustainability information.

In addition to the laboratory testing data, in-depth nutritional analysis and scientific reports were prepared by the SAT on subjects including nutritional reference intakes, dairy fats, plant-based oils, omega-3 fatty acids, pre- and probiotics, sodium limitations, sugar limitations, heavy metals, choline, coconut oil, protein, electrolytes, fiber, and food additives (natural and artificial preservatives, sweeteners, flavor enhancers, thickening agents, emulsifiers, anti-oxidants, synthetic and natural colorants). These targeted reports helped both in terms of narrowing the focus for testing and interpreting the test data. The SAT conducted extensive literature reviews, paying mind to corporate sponsorship, to support each component and threshold.

We employed the *Eurofins* Nutrition Analysis Center (Des Moines, IA) who conducted comprehensive testing of products and ingredients. The SAT developed a comprehensive list of ingredients and products for testing, including dairy components, fats, juice concentrates, emulsifiers, stabilizers, and yogurt cultures, in addition to selected whole foods (plant and animal-based) and finished products (e.g., culinary, juice, dairy). Specific *Eurofins* Tests are found in Table 3 and test methods of reference were explicit in all tests, and international shipping of all substances required logistical planning.

**Table 3:** Product and Ingredient Testing (Eurofins).

Macronutrients	Vitamins	Micronutrients	Heavy metals	Other
Omega-3 polyunsaturated fatty acids	Vitamin A (Retinol)	Choline	Lead	Anthocyanins
Saturated Fats	Vitamin A (Beta Carotene)	Sodium	Arsenic	Polyphenols
Omega-6 polyunsaturated fatty acids	Vitamin D	Copper	Cadium	Flavonoids
Transfats	Vitamin E (Tocopherol Profile)	Magnesium	Mercury	Glyphosate
Total protein	Vitamin C (Ascorbic Acid)	Maganese		Colony Forming Units (CFUs)
Amino acid profile	Vitamin B1 (Thiamine)	Iodine		Juice Authenticity
Sugar profile	Vitamin B2 (Riboflavin)	Iron		
Fiber profile	Niacin	Phosphorus		
	Vitamin B5 (Pantothenic Acid)	Potassium		
	Vitamin B6 (Pyridoxine)	Selenium		
	Vitamin B12	Zinc		
	Total folate	Calcium		

Three tiers provide a measured application of change across the consumer packaged goods industry. All tiers are “as is” assessments. Any product in any tier can be reclassified after that product has been re-engineered. The tiers are progressive, moving from the lowest ranking to best in class. Each tier includes an overview, assumptions, criteria, and references. The tiers provide a tool for the Re-engineering Team to triage and provide a transparent guide for applying the Matrix to specific products.

#### 2.5.2. Perfact

All the test results data from Eurofins were entered into the Genesis R&D database, along with other sources of data, then exported *via* integrated JSON files to the *Perfact* food data analysis system, which permits complex filtering based on specific criteria and provides focused and actionable information in a strictly declarative manner, designed to describe metabolic health and environmental impacts.

The Metabolic Matrix applies an advanced set of criterion types supported by the *Perfact* food data science platform. These criteria can range in complexity, such as “no added sugar” or a threshold for specific types of sugars (e.g., sucrose, or specific targets for its subcomponents glucose and fructose). Ingredient criteria that consider allergens, toxins and toxicants, sourcing (traceability, GMOs, etc.), macro- and micro-nutrient preferences (with specific amounts, thresholds, and ratios), and types of processing are also applied (e.g., cold-pressed oils).

The core analytical engine can ingest data from any number of structured data sources such as XML, HTML, CSV, JSON, etc. Depending on the application context, output can be per-product evaluations and – if a body of evaluated products exist – recommendations based on any number of criteria complete with a full breakdown of why a product was recommended or not. Beyond the modules for ingestion, analytics, reporting, and recommendation, the platform consists of additional modules for ingredient documentation, web- and app-based user access, as well as APIs for online retail integration – all of which go beyond the scope of this paper.

Basic criteria include the established nutrient thresholds based on serving sizes or reference units (e.g., the FDA’s “low sodium” criteria), thresholds for ratings (e.g., Environmental Working Group’s “minimally processed score”), presence on inclusion/exclusion lists (e.g., FDA product recalls), and the presence/absence of ingredients. This is because ingredient statements can be found in numerous forms and levels of detail. For example, the engine recognizes different spellings (citric acid will be recognized in different forms such as 2-hydroxypropane-1,2,3-tricarboxylic acid, acidum citricum, or E330), common misspellings, group names (glycerol monolaurate, monolaurin, 1-lauroyl-glycerol, etc., will be classified as instances of mono- and diglycerides of fatty acids) and steers clear of verbatim matching that might consider a peanut a nut because it contains the letters “nut.” Complex criteria allow differentiation by category, ingredient concern confidence level and ingredient modifiers (“organic”) which can be arbitrarily combined with any basic criteria. For example, for non-GMO criteria set, citric acid could be declared as a potentially offending ingredient (instead of an offending ingredient) which can be neutralized by either a prefix indicating non-GMO status (such as “non-genetically engineered,” “without GMO’s,” “naturally derived,” etc.) or the product’s identifier being present on a whitelist. Finally, calculated ratings can be employed to implement any rating system such as the UK FSA’s traffic lights, Nordic keyhole, NutriScore, as well as the tiered approach described here.

Data from various departments (product development, production, sales, packaging, sustainability) were consolidated by KDD’s IT department into a single JSON file for each product (e.g., strawberry yogurt, chocolate milk, etc.) and each product component (e.g., milk, butter, water, etc.). The single file approach minimized the number of data exchange interfaces and allowed high-level syntax and consistency checks by KDD before export by means of a validator tool provided by *Perfact.*

Results from the evaluation comprising over 75,000 data points were reported in two forms. The first was a report consisting of one page per product that documented how each product did or did not meet each criterion, the product’s tier rating, and a set of recommended modifications for a product to reach the next highest tier. For economic reasons, not all assays were carried out for all product components. Therefore, this report also includes a set of assumptions that were made, for example, by drawing upon a reference nutrient for grape juice if grape juice was not fully tested. Secondly, we presented an overview spreadsheet that combined input data and all data from the per-product reports minus recommendations and assumptions.

With these reports, KDD had a solid base for deciding whether to keep, re-engineer, or discontinue a product. Because the report clearly lists recommended modifications, KDD was able to identify “easy win” products that just require minimal modifications to ameliorate current shortcomings. Also, with the engine fully set up after the first round of evaluations, KDD’s product development team can now have *ad hoc* evaluation runs of projected products to get recommendations on how to improve them before the complex production process has been started.

Finally, the systematic validation of existing component data has revealed many opportunities for component vendors to improve their specification sheets. For example, KDD could opt to require specification sheets to be provided as data files (instead of as PDF documents) which would need to pass validation steps before being incorporated into KDD’s component pool. Since Tier I criteria included evaluation concerning the presence of “Big 8” allergens (milk, eggs, fish, shellfish, tree nuts, peanuts, wheat, and soybeans; which will be “Big 9” after the addition of sesame by the FDA effective 2023), KDD can easily enhance product labeling by highlighting allergens and thus stand out against competitors by surpassing existing MENA labeling standards.

## 3. Results

During 2020 and 2021, Phase I of the KDD Metabolic Re-engineering Initiative involved the development of the Metabolic Matrix and its quantified expression. Phase I included multiple teams: SAT, Product Reengineering Team, Product Development, Ingredients Committee, Juice Taskforce, Marketing, Sales, IT, and Senior Leadership. The criteria in TIERS classification, including 38 criteria organized into five progressive levels, were programmed into *Perfact*. The comprehensive report was supported by dozens of specific technical reports prepared by the SAT on topics related to the TIERS criteria.

**Figure 1:**
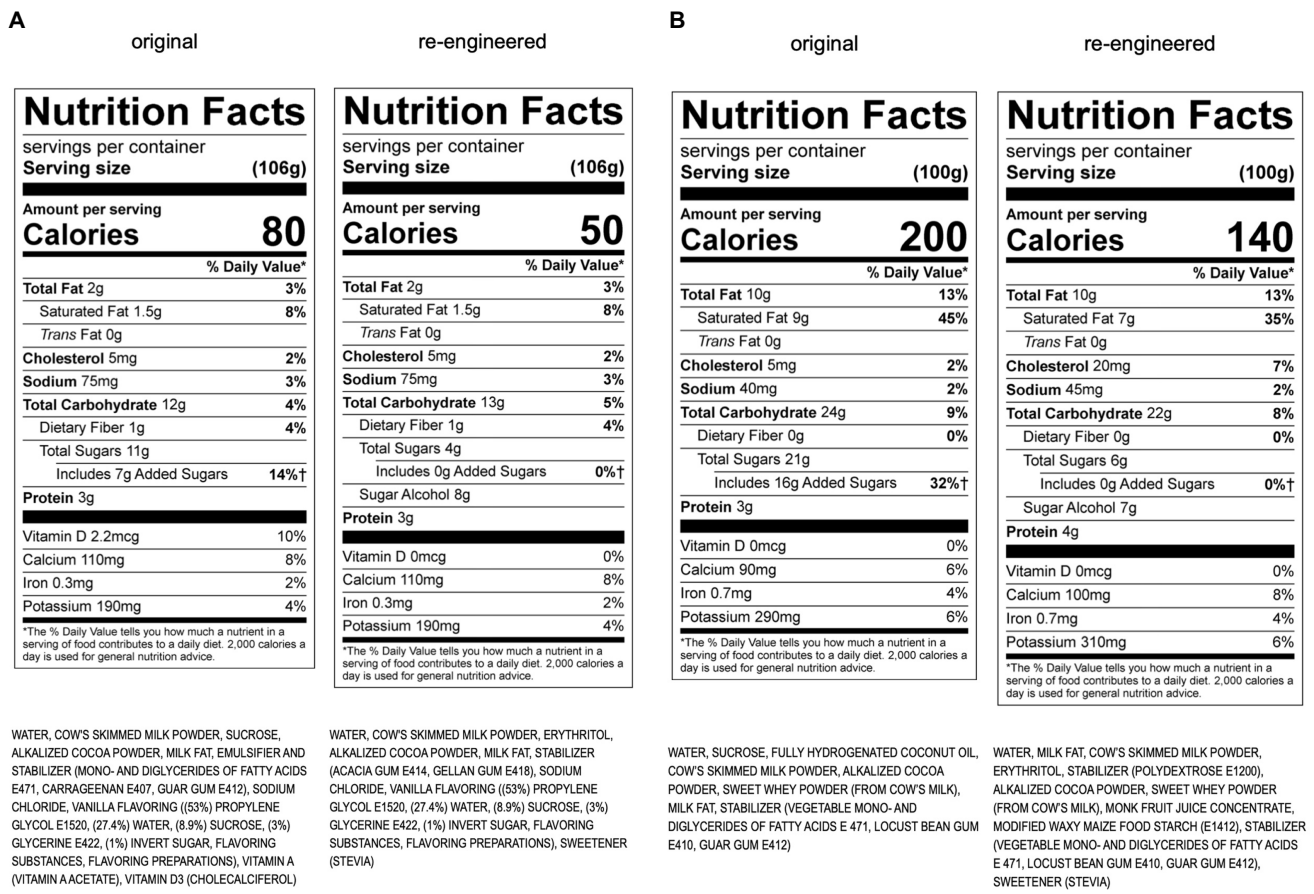
**(A)** Re-engineering of KDD chocolate milk using the Metabolic Matrix. **(B)** Re-engineering of KDD chocolate ice cream using the Metabolic Matrix.

During Phase II, a Senior Leadership Strategy Team was assembled to interpret, prioritize, and translate the prolific amount of Phase I information and recommendations presented by the SAT and *Perfact*. Priority targets, action plans, and work streams were established, using a matrix to prioritize efforts based on maximum impact (e.g., volume, sales/profitability, metabolic impacts, and key product groups: dairy, ice cream, juice, and culinary). Four critical areas of focus across the entire portfolio were identified: sugar, fiber, omega-3 s, and emulsifiers/stabilizers. Re-engineering projects set into motion include both existing products and new product development (see example of chocolate milk in Figure 1A and chocolate ice cream in Figure 1B). It became evident in Phase II that many new types of ingredients are needed to reengineer products for optimal metabolic effects, so the development of an additives matrix to provide a rigorous scientific approach to evaluating all currently used food additives was set in motion.

The TIERS classification is a working model and not a static framework and is subject to modification as the science evolves and is tempered with the realities of what is involved with implementing the criteria at an industrial scale. It has also evident that the TIERS classification is not a “one-size-fits-all” approach. It must be adapted to be relevant to broad product groups (e.g., dairy, juice, etc.) and specific product types. There are many challenges where adaptation is needed (shelf life, regulatory limitations, availability of ingredients, cost factors, processing technology, consumer acceptance, etc.).

## 4. Discussion

By integrating the science of metabolic health with industrial concerns and procedures, we have sought to provide an evidence-based multidisciplinary strategy to address the chronic metabolic disease pandemic engendered by our ultraprocessed food supply. Based on morbidity, mortality, and economic cost, noncommunicable diseases (NCD) are the second most important global health problem currently facing humanity (the first being climate change) (75). While engaging in this exercise, we remained cognizant of the overriding concern that we must be able to feed 10 billion people by the year 2050. Processed food is not going away: indeed, it will be required. The conundrum is how to meet these two dichotomous priorities simultaneously. Our mission in re-imagining the KDD portfolio was to use the evidence-based literature to make processed food healthier, and our strategy was to adhere to the Metabolic Matrix of *protect the liver, feed the gut, and support the brain*.

Ultraprocessed foods are the product of the post-World War II era when issues of mass food production, security, safety, shelf-life, and profit were dominant. Unfortunately, they have since fuelled the pernicious aftermath of global increases in obesity, type 2 diabetes, and other NCDs, for which modern medicine cannot provide an effective antidote (76). The data have clearly demonstrated that ultraprocessed foods are detrimental to human health due to numerous excesses, including trans-fats, sugar, branched-chain amino acids, omega-6 fatty acids, emulsifiers, additives, salt, antibiotics, and nitrates. In addition, ultraprocessed foods are creating havoc with the biochemistry of the brain. Children are especially vulnerable to specific nutritional insufficiencies, including inadequate omega-3 essential fatty acids, a lack of gut-beneficial prebiotic dietary fiber leading to limited serotonin production, and a lack of key nutrients vital for neurotransmitter function, cognition, mood, sleep, and optimal neurodevelopmental outcomes (21, 77).

Environmental obesogens are now implicated as one of several primary causes of obesity and chronic disease (6). An obesogen is defined as an endocrine-disrupting chemical that alters energy balance by binding to cellular receptors to promote adiposity greater than its caloric equivalence (78). Based on this definition, ultraprocessed foods meet these criteria. A recent randomized crossover trial demonstrated that the same macronutrient and micronutrient composition provided as ultraprocessed food led to differential weight gain compared to unprocessed food (79). Several components comprising ultraprocessed foods qualify as metabolic toxins based on mechanisms of action, including but not limited to:

- *Mitochondrial dysfunction.* Fructose inhibits three mitochondrial enzymes involved in ATP generation — AMP kinase, acyl CoA dehydrogenase long-chain, and carnitine palmitoyl transferase-1 (80).
- *De novo lipogenesis*. Fructose is a preferred hepatic lipogenic substrate driving liver fat accumulation and insulin resistance (81). In addition, the essential branched-chain amino acids (BCAA’s) leucine, isoleucine, and valine contribute to fatty liver disease as well (81). BCAA’s are found in higher amounts in corn-fed beef, chicken, and fish; all processed food staples (82).
- *Insulin secretion*. Despite their lack of calories, diet sweeteners increase insulin secretion (83) and have been shown to correlate with all-cause mortality (84).
- *Insulin sensitivity.* Inadequate fiber intake is associated with reduced insulin sensitivity (85), and all-cause mortality (40).
- *Altered microbiome and inflammation*. Lack of fiber is also associated with microbiome changes consistent with altered metabolic health (86). Furthermore, commercial emulsifiers such as carboxymethylcellulose are associated with intestinal inflammation (87) and certain non-nutritive sweeteners have recently been shown to consistently alter the human microbiome, resulting in glucose intolerance (88).
- *Fat cell differentiation*. Chemicals involved in food processing and packaging, such as bisphenol-A, are known to activate peroxisome proliferation-activated receptor-gamma (PPARγ) (89), which can stimulate adipocyte differentiation and proliferation. Insecticides such as chlorpyrifos or neonicotinoids, preservatives such as parabens, and antioxidants such as 3-tert-butyl-4-hydroxyanisole (3-BHA) are also potential obesogens (6).

### 4.1. Strategies

Despite these serious health concerns, the consumption of ultraprocessed food is not declining (90). Production and sales continue to increase globally (91) and are likely to continue if food commodities (such as corn, wheat, soy, and sugar) are subsidized by various governments to make ultraprocessed food cheap and readily available. Therefore, it is imperative to ameliorate the metabolic perturbations that negatively impact physical and mental health. Our efforts can be categorized along four paths of intervention:

#### 4.1.1. Sugar reduction

Based on mechanistic, clinical, and epidemiological data, sugar reduction was a primary directive of our re-engineering effort. Many options were considered, including enzymatic breakdown of fructose, filtering out of sugar, and caramelization. Ultimately, we settled on an upper limit of one teaspoon of sugar per serving, to maintain sweetness and stimulate reward and sales, while reducing metabolic detriment. One item in KDD’s portfolio is juice. Solving the problem of the high sugar content in juice is challenging, but the range of strategies that are being applied are listed in Table 4.

**Table 4:** Mitigation strategies for sugars in juice.

Strategies	Description
No added sugars	Do not add sugar to products already containing high amounts of sugar
Microbial technology	Use of organisms to consume the sugar
Nanofiltration	Filtering out sugar
Fiber	Mitigating the impact of fructose with whole fiber
Modify juice to water ratio	Less juice or juice concentrate, more water
Replace sugar with non-caloric sweeteners	Use of non-caloric sweeteners
Investigate new sugars and sweetener solutions	Evaluate and adopt new solutions such as allulose, tagatose, protein-based sweeteners, sweetener combinations, etc.
Modifying ingredients	Modulators, enhancers, texturizing agents
Portion control	Use smaller containers
Branding and marketing	Highlight positive effects of low/no sugar-added products
Public health education	Emphasizing the importance of reducing sugar
Packaging/labeling	Highlighting positive health attributes of low or no sugar foods and beverages
Policy	Sugar tax, remove subsidies on sugar, subsidize healthy solutions, and fast track regulations to adopt new alternatives to sugar

Food and beverage companies are under increasing pressure to reduce or eliminate added sugar as consumers seek to avoid consuming ‘empty calories’ that drive metabolic disease (92). Many investors with little experience in the beverage industry are moving into the fruit-flavored beverage space, attracting the attention of the largest beverage companies. There are many ingredient solution options available in the burgeoning market, as ingredient supply companies are providing solutions to create healthier sugar-reduced, no-added-sugar (NAS), or sugar-free options that provide nutritional benefits. KDD is applying the Metabolic Matrix developed by the SAT to develop a range of no-added sugar products, including both dairy and juice-flavored beverages, and currently has numerous NAS products in the development pipeline. Solutions include diluting or replacing juice with water, adding other ingredients such as juice flavors, oils, essences, sweeteners, and other functional inclusions such as herbs, flowers, spices, vitamins, minerals, probiotics and prebiotics (fiber). Indeed, the fruit-flavored and functional beverage markets are growing rapidly on a global scale.

#### 4.1.2. Ingredients

##### 4.1.2.1. Non-nutritive sweeteners

Using the available literature, we evaluated the metabolic plausibility, feasibility, and efficacy of substituting low or no calorie sugars/sweeteners to replace sucrose (93). Two competing issues had to be resolved. First was the possibility that non-nutritive sweeteners possess their own morbidity unrelated to calories (88, 94). Second is the difference in the CNS reward signal between glucose, fructose, and non-nutritive sweeteners (95) as sales are dependent on maintenance of reward. Furthermore, we analyzed the metabolic profiles and price points of all current non-nutritive sweeteners, as well as the current political climate surrounding European Food Safety Administration (EFSA) and Gulf Cooperation Council Standardization Organization (GSO) approvals.

A thorough literature review of alternatives to sugar resulted in a short list of substances that are also approved for use by the GSO. The GSO tends to wait on the European Food Safety Authority, following their lead in terms of approving substances. While regulation of sugar seems to be of little concern to the GSO, sweeteners, on the other hand, are treated with suspicion and require warning labels on the products using them.

The process of selecting alternatives to sugar was first and foremost based on the metabolic impacts of these substances. Various solutions are needed, as sugar has many properties valued in food processing above and beyond providing sweetness (crystalline structure, mouthfeel, preservative, flexibility in multiple types of food and beverage formulations, exposure to heat and pressure, etc.). In many cases, variations and combinations of different types of sweeteners are required to achieve a balance of sweetness and those formulations are often specific to the type of food product involved.

Many non-caloric sweeteners have been linked to health concerns, and there is no “one size fits all” sweetener solution. Allulose emerged as the optimal solution for a variety of reasons related to evidence-based positive metabolic impacts, but the substance is not yet approved for use by the GSO, which appears to be waiting to see what EFSA does, and indications are EFSA may not approve the substance until 2024. In the meantime, the shortlist of sweeteners identified with the least harmful and most positive metabolic effects are erythritol, stevia, and monk fruit juice concentrate. These substances are well researched, offer a wide variety of formulations, and are abundantly available on a commercial scale.

##### 4.1.2.2. Fats

The human brain has specific dietary needs and the association between the types of foods we consume and mental health is an area of rigorous investigation by global leaders in the field of nutritional psychiatry. Our focus on supporting the brain is based on the knowledge that the brain is an organ with biological and chemical underpinnings.

The brain comprises specialized, unique, and complex lipids and around 25% of all neuronal membranes are made up of DHA. Omega-3 fats perform a wide range of significant biological roles throughout the central nervous system, e.g., reducing inflammation and facilitating neurotransmitter function, as well as assisting in the regulation of key hormones required for mental health, such as serotonin and dopamine.

ALA is a plant-based, omega-3 polyunsaturated essential fatty acid that is only available *via* the diet and is present in certain nuts, seeds, and green leafy vegetables. The two key highly unsaturated omega-3 fatty acids in the brain are DHA and EPA, which are sourced from oily fish and seafood. DHA plays a key role in neurotransmitter function, and EPA has anti-inflammatory effects. Both are brain-essential and generate neuroprotective metabolites. EPA has been clinically demonstrated to reduce attention deficits and depression in human populations. It is recommended that adults should be consuming between 250 and 500 mg of EPA and DHA daily, although this recommendation differs within clinical populations (96). Paleolithic diets of our ancesters have estimated ratios of omega-6 to omega-3 in the region of 1:1 to 4:1, based on current and past evidence we proposed an omega-6/3 target ratio in the TIERS document, of no more than 4:1 (97).

Our mission of supporting the brain requires KDD products to contain healthy (unrefined) brain-essential fats, and simultaneously reduce and/or eliminate intakes of industrially produced (hydrogenated) and pro-inflammatory seed oils (e.g., soybean oil). We have targeted the promotion and inclusion of healthy fats, namely (i) monounsaturated fatty acids (MUFAs), (ii) polyunsaturated fatty acids (PUFAs) e.g., plant-based, short-chain dietary sources of ALA, and (iii) marine-based or algal sources of long-chain, omega-3, highly unsaturated fatty acids DHA and EPA, which have the potential for metabolic health improvement. Where possible, we fortified KDD products with EPA and DHA by way of algal and/or marine oils. To meet these objectives, we first analyzed the omega-6 (seed oils), omega-3, and *trans*-fat content of all KDD products to assess which products contained a less than optimum omega-6 to omega-3 ratio of 4:1. We also summed the combined total of EPA and DHA and calculated which products had elevated amounts of omega-6 and zero or low omega-3. We measured artificial *trans*-fat content with a view of total elimination. We recommended shifting away from partially hydrogenated forms of coconut oil (a popular functional fat used in commercial products) and replacing these with extra-virgin, unrefined bioactive vegetable oils such as olive oil. We also provided options for fortification and recommendations to add specific nutrients, e.g., fatty acids and vitamins that are supportive of metabolic and brain health and function (98, 99).

##### 4.1.2.3. Fiber

To optimize feeding the gut, we had to optimize both prebiotics and probiotics. The clearest path to the former is through both soluble and insoluble dietary fiber. This demands a collaborative effort between the SAT and KDD leadership to select existing and novel ingredients that can achieve these aims and able to enhance product stability, palatability, mouthfeel, and satiety. For example, we replaced carrageenan with alternative soluble fibers that can reduce potential harm while maintaining product texture and flavors.

##### 4.1.2.4. Dairy-based ingredients

We carefully reviewed evidence from a range of current, credible, scientific sources which demonstrated that high-fat diets are not robust predictors of obesity or weight loss as previously thought. Emerging research indicates dairy saturated fats are indeed protective against NCDs (100). Rather, it is high intakes of refined carbohydrates and sugar in processed foods that play a primary role in the epidemic of diabetes and the potential development of cardiovascular disease. We further support the notion that calorie counting only has transient effects in relation to weight loss in terms of metabolic health, and that contrary to popular belief, diets higher in fat, especially unsaturated cis-fatty acids (e.g., Mediterranean and ketogenic diets) (101, 102), are likely beneficial. Type 2 diabetes is considered by and large preventable, and the simplest modifiable factor is dietary alterations (e.g., sugar and carbohydrate reduction). Critically, the latest research does not support the diet-heart hypothesis of the 1970s in terms of cholesterol being the sole driver of cardiovascular disease (103, 104): the story is just not that simple (104). Furthermore, low-fat diets have not been shown to lead to weight loss necessarily, and novel models such as the Carbohydrate-Insulin Model (CIM) of obesity are growing in popularity (105). In addition, recommendations to limit whole-fat milk are arguably outdated and lack scientific credibility, given new evidence that full-fat dairy may be beneficial in terms of lowering disease risk for metabolic syndrome (106–108) and type 2 diabetes (109–111).

The SAT provided novel methods to optimally enrich KDD milk and ice cream products with omega-3 fats (EPA and/or DHA), which meet our criteria for supporting the brain. We conducted rigorous scientific literature reviews of macronutrients with a focus on dairy products. We critiqued the low-fat versus full-fat dairy debate before settling on recommendations for the inclusion of whole fat milk from pasture-raised ruminants containing LC-PUFAs, ALA. We advised fortifying the milk with fat soluble vitamins (e.g., vitamins A and D) and trace elements (such as zinc, magnesium, iron).

##### 4.1.2.5. Processing

New equipment and technology are an inevitable aspect of this initiative, and this requires significant capital expense. KDD has installed a new filling and sealing line that allows fruit and other inclusions to be included in yogurt, allowing for additional ways to supplement yogurt with whole food ingredients.

##### 4.1.2.6. Packaging

Portion sizes on the pack have long been problematic. For example, a pint (473 mL) of ice cream may claim that it contains four servings, presumably meant to be eaten on four separate occasions or by four different people, but this is not necessarily what consumers eat. In many countries, regulations require serving sizes to be based on how much a person will realistically consume versus what is prescribed by nutrition authorities or stated by manufacturers. One packaging trend in Kuwait has been to use increasingly large containers for foods and beverages. An approach to mitigation might be to offer juice in smaller quantities using well-marketed concepts such as “juice shots.”

##### 4.1.2.7. Food data science

Given the parameters defined by this endeavor, picking a data science strategy had to meet three requirements. First, we must accommodate complex, tiered criteria that go beyond plain numerical nutrient data and includes a robust treatment of ingredients. Second, we must deal with data from a multitude of sources with overlapping structures for the same product. For example, products that are made of several levels of components which only in turn comprise ingredients; or packaging data that is independent of internal product composition; or vendor-specific certifications; or pricing information. And third, we must produce precise, fully traceable results.

Reviewing the available Food Data Science literature, we found that Food Data Science at its core is not driven by food data, but rather to use a reductionist approach to make predictions that strips away food-specific properties (e.g., Machine Learning). While such interpretation of Food Data Science has its place, the focus of our work was not about making predictions, but rather evaluations. Even if we had tried to construe evaluation as a special form of prediction, the nature of neural networks precludes the precise traceability of results, because after training the neural network yields results without giving an explanation that can be submitted to scrutiny. Also, we did not find a single description of a computational Food Data Science method that safely dealt with textual ingredient information (as opposed to numerical nutrient information) at a sufficiently high confidence level.

Even though the existing results of Food Data Science did not fully meet our requirements, we still felt that our effort fell into those confines and should be called by that name. We encourage expanding its methodological scope to include approaches that focus on the idiosyncrasies of food data first, and only then choose the appropriate method based on the relevant task at hand.

The *Perfact* platform met our evaluation requirements. Due to its declarative nature of defining criteria, changes formulated by the SAT could be applied right away to all KDD products without necessitating programming or training times. This proved to be a valuable quality when integrating evaluations of projected products into the product design cycle.

#### 4.1.3. Global implications

From an engineering standpoint, ultraprocessed food is mass produced, is consistent batch to batch, is consistent country to country, uses specialized ingredients from specialized companies, consists of pre-frozen macronutrients, stays emulsified, and has long shelf life or freezer life (21). While ultraprocessed foods are good vehicles for adding micronutrients, especially in geographic areas with endemic deficiencies, it is nonetheless clear that ultraprocessed food components, through the metabolic consequences noted above, lead to inflammation, insulin resistance, and chronic metabolic disease. However, this also affords an opportunity to improve the health of the population at large. This is exemplified by the partnership between the United Kingdom and CPG companies in the early 2000’s to decrease the salt content of ultraprocessed food by 30%, resulting in a 40% reduction in the prevalence of hypertension and stroke (112). Regions like the MENA suffer disproportionately from food-related diseases, such as obesity, diabetes, cardiovascular disease, and osteoporosis (113) in part due to ultraprocessed food consumption. If multiple companies adopted the Metabolic Matrix precepts, we anticipate that the region would similarly experience a reduction in these diseases.

### 4.2. Market forces

Flavor is one of the most critical aspects for effective marketing. Labeling products as “healthy” sets up an expectation that the product will not taste as good as the “traditional” formulation, and is counterproductive to product sales. However, long-term consumer attitudes can be improved simply by using healthier ingredients. Creating delicious products for consumers that are also good for them is critical.

With respect to sugar and fructose reduction, there are significant challenges with respect to the current costs of non-nutritive sweeteners. Currently, the cost differential is dramatic with the current retail cost of sucrose at 0.11 cents per teaspoon. The equivalent sweetness of allulose, monkfruit, and erythritol are ~9.3 cents, 2.5 cents and 4.6 cents per equivalent serving. While that cost differential will have a significant impact, costs have been narrowing dramatically in the last 3 years with multinational sweetener firms racing to bring cost effective non-nutritive sweeteners to market.

### 4.3. Strengths and limitations

The framework of the Metabolic Matrix provides a clear rationale and roadmap for other food companies to modify the healthfulness of their products. By focusing on five food parameters: sugar reduction, fiber supplementation, healthy fats, micronutrient addition, and adulteration negation, any company can offer improved products that are metabolically beneficial. Our work is evidence-based, aligned with both health and industrial directives, achievable in a short period of time with only marginal cost of implementation, and results in a marginal increase in price point for the consumer. Engaging in this exercise also increases transparency, ethics, documentation of the origin of food ingredients, and labeling. It can also elaborate a more defensible commitment to environment, sustainability, and governance for the public.

We recognize that, even while relying on scientific evidence from the literature, we have made many assumptions, including those on sugar alternatives, omega-3 fatty acids, stabilizers, preservatives, and emulsifiers. Clearly more research is needed on allulose, tagatose, and various dietary fibers. We have not performed either supply chain or economic modeling to demonstrate the cost effectiveness or environmental sustainability of this approach at an industrial level. Also, while we (114) and others (115) have previously performed microsimulation analyses to demonstrate the health and healthcare benefits of sugar reduction, a similar analysis on ultraprocessed foods has not yet been done. Lastly, we believe that employing the Metabolic Matrix will save governments healthcare costs resulting from the premature development of NCD’s.

## 5. Conclusion

The balance sheet between the economic benefits versus the healthcare costs of ultraprocessed food is markedly disproportionate, and is rapidly depleting governmental healthcare budgets worldwide. CPG companies have historically either been unwilling or financially unable to address the metabolic detriments of their products, in part due to net corporate profit and in part because of fear of consumer backlash. We have engaged in this exercise to find an alternative path forward for KDD, one that can be implemented by other companies.

Re-engineering food for metabolic health requires more than reformulation. It requires a new business model that supports multiple bottom lines, champions human and environmental health, and creates entirely new market segments for novel or reformulated products to gain traction in the marketplace. Optimizing products for metabolic health impact requires firm resolve and leadership from commercial interests to activate change on multiple fronts: government, academia, public health, and NGOs. Any food company seeking to innovate and build a metabolically healthy portfolio is currently participating on an uneven playing field.

Virtually every ingredient and processing solution advancing metabolic health currently requires additional cost. Non-nutritive sweeteners can cost 7–10 times as much as sucrose and high fructose corn syrup, and new types of processing which maximize metabolic health effects in foods and beverages may require significant capital investment. Reducing the commercial use of sugar requires novel incentive structures and ingredient solutions. These, however, are sorely lacking and require governments and global markets to enable metabolically supportive and economically viable alternatives to sugar. Regulations in the MENA region also do not encourage innovation to optimize for metabolic health. For example, Saudi Arabia taxes both sugar and non-nutritive sweeteners to derive revenue (versus health impact), thereby disincentivizing commercial efforts to provide healthier alternatives with no added sugar. Some MENA countries also allow imports of highly processed foods manufactured by global food and beverage corporations with unregulated pricing, while at the same time forcing companies in-country to maintain artificially low prices on their products, with no incentives for companies seeking to improve the metabolic impact of their products.

Ultraprocessed foods are only “cheap” when the costs of their negative metabolic impact are externalized to health care and public health budgets. The burden of internalizing the real costs of food and making products that improve metabolic health is currently shouldered by individual companies. Cooperation is fundamental to food system change: no company can solve global health challenges independently, so the whole system needs to change (116). Therefore, we have worked with KDD to share this re-engineering approach with the global community.

If food is the equivalent of medicine (117), then food companies are akin to physicians. The public trusts their doctors to have their best interests at heart, or at least to live up to their pledge: *primum non nocere* (first do no harm). CPG companies similarly must agree to mitigate the metabolic harm of their products as a primary goal. We offer the Metabolic Matrix — *feed the gut, protect the liver, support the brain —* as a roadmap to safeguard their mission, reputation, profits, and global consumers.

## Data availability statement

The original contributions presented in the study are included in the article/Supplementary material, further inquiries can be directed to the corresponding author.

## Author contributions

All authors listed have made a substantial, direct, and intellectual contribution to the work and approved it for publication.

## Funding

This study was supported by Robert H. Lustig Research Foundation.

## Conflict of interest

RL is Chief Medical Officer of BioLumen Technologies, Kalin Health, Perfact, and Foogal, and a paid advisor for ReadOut Health, Levels Health, Simplex Health, and Myka Labs. AK is Chief Executive Officer of Perfact. PA is Executive Manager, Human and Environmental Health at KDD, and Chief Commercial Officer of Perfact.

The remaining authors declare that the research was conducted in the absence of any commercial or financial relationships that could be construed as a potential conflict of interest.

## Publisher’s note

All claims expressed in this article are solely those of the authors and do not necessarily represent those of their affiliated organizations, or those of the publisher, the editors and the reviewers. Any product that may be evaluated in this article, or claim that may be made by its manufacturer, is not guaranteed or endorsed by the publisher.
