# Supplementary material for: The Metabolic Matrix: Re-engineering ultraprocessed foods to feed the gut, protect the liver, and support the brain
Source: Front Nutr. 2023 Mar 30;10:1098453. doi: 10.3389/fnut.2023.1098453 (PMC10097968; doi:10.3389/fnut.2023.1098453)
Supplement: Supplementary file 1 [file Data_Sheet_1.pdf]

Supplementary Tables
List of References for information is Supplemantary Tables A - H

Supplementary Table A. Food Additives. Synthetic and natural colorants which may have potentially harmful side-effects.

Synthetic Colorants	Food Additive	Not Approved for use in the following countries	Potential links to the following harms
E102	Tartrazine / FD&C Yellow 5	Norway, Austria	Allergies, hyperactivity, thought to be contaminated with benzidine or other carcinogens, may be detrimental to the development of tumors and other cancer, may be linked to white blood cell damage
E104	Quinoline Yellow / FD&C Yellow 10	Norway, USA, Austria and Japan	Hyperacitivty and behavioral effects in children
E106	Riboflavin-5-sodium phosphate	None	Eye health, headaches
E107	Yellow 2G	Norway, USA, Japan, Sweeden, Switzerland	Allergic reactions, intolerant among asthmatics and those with an aspirin intolerance
E110	Sunset Yellow FCF / FD&C Yellow 6	Norway, Sweden, Austria, Switzerland, Japan and USA	Allergic and intolerant reactions, nasal congestion, hives, abdominal pain, nausea, and GI complaints, migranes, hormonal changes, kidney tumors, swelling of skin, hyperactivity in children
E122	Azorubine / Food Red 3	USA and Sweden	Rashes (hives), sensitivity by those with asthma, allergic reactions such as swelling of the skin. May cause hyperactivity and water retention
E123	Amaranth	Norway and USA	Suspected carcinogen which is linked to cancer
E124	Ponceau 4R	Norway and USA	Links to cancer and hyperactivity in children
E127	Erythrosine / FD&C Red 3	EU and UK (restricted use, permitted in cocktail and candied cherries)	Links to thyroid toxicity, possible links to inattention and hyperactivity in children
E128	Red 2G	Norway, USA, Japan, Sweeden, Switzerland, Austria and Australia	May interfere with blood haemoglobin and it is not recommended for consumption in children
E129	Allura Red AC / FD&C Red 40	Denmark, Belgium, France, Germany, Switzerland, Sweeden and Austria	Linked to allergies e.g., skin irritation, sneezing, watery eyes, facial swelling, migraines and behaviour symptoms such as aggression and ADHD-type symptoms in children
E131	Patent Blue V	Norway, Australia and the USA	It is associated with allergic reactions ranging from mild urticarial rash (grade 1) to severe anaphylaxis
E133	Brilliant Blue FCF / FD&C Blue 1	Austria, Belgium, Denmark, France, Germany, Greece, Italy, Norway, Spain, Sweden and Switzerland.	Linked to asthma, hyperactivity in children, tumors and cancer
E142	Green S	Canada, United States, Japan, and Norway	Allergic reactions which can be severe when directly exposed to the skin, hyperactivity in children, tumors, GI complaints including diarrhea, nausea, vomiting and bloating
E143	Fast Green FCF / FD&C Green 3	EU	Potential tumorigenic effects in experimental animals, as well as mutagenic effects in both experimental animals and humans. It has associated risks in terms of irritation to the eyes, skin, digestive tract, and respiratory tract in its undiluted form
E151	Brilliant Black BN	United States, Switzerland and Japan	Allergies, intensified asthma, hyperactivity and ADHD in children, digestive problems, cancer
E154	Brown FK	EU, Australia, Austria, Canada, Finland, Ireland, Japan, Norway, Sweden and USA	Exacerbates asthma especially in combination with benzoates. Linked to hyperactivity in children
E155	Brown HAT	Australia, Austria, Belgium, Denmark, France, Germany, Norway, Sweden, Switzerland and USA	Allergic reactions especially in asthmatics and those sensitive to aspirin, may cause skin irritations
E180	Lithol Rubine BK	Australia	Not recommended for people who have an intolerance to salicylates. May worsen asthma and yield negative effects to those who suffer from rhinitis or the skin disease urticaria. Linked to hyperactivity in children
**Natural Colorants**			
E150a-d	Caramel	None	May contain potentially carcinogenic residues when processed with amonia-based colorants. The European Food Safety Authority (EFSA) has concluded that caramel colours are not carcinogenic or genotoxic
E153	Carbon	USA	Suspected as a carcinogenic agent. Linked to otential side effects including constipation, and in more serious, but rare cases, it may cause slowing or even blockage of the intestinal tract and dehydration
E161a	Flavoxanthin	USA and EU	Investigations of the safety of fucoxanthin consumption in humans is lacking
E161d	Rubixanthin	USA and EU	Less than 5 safety studies in humans
E161e	Violaxanthin	USA and EU	None known. Safety studies in humans are lacking
E161f	Rhodoxanthin	USA and EU	None known. Safety studies in humans are lacking
E161g	Canthaxanthin	Australia and New Zealand	May cause diarrhea, nausea, stomach cramps, dry and itchy skin, hives, orange or red body secretions, and other side effects. Safety studies in humans are lacking
E171	Titanium dioxide	EU	Potential genotoxicity links
E172	Iron oxides	Germany	Possible kidney damage. Suspected neurotoxin. Blindness in dog studies.
E173	Aluminum	None	Large amounts or prolonged use are linked in experimental animal studies to skeletal deformations , Alzheimer's Disease and osteoporosis in animals
E174	Silver	Australia	Regular consumption may be linked to kidney damage and a blue-grey discoloration of the eyes, nose and nasal septum, throat and skin

Supplementary Table A References. Food Additives. Synthetic and natural colorants which may have potentially harmful side-effects.

1. Center for Science in the Public Interest. Food dyes: a rainbow of risks. https://www.cspinet.org/sites/default/files/attachment/food-dyes-rainbow-of-risks.pdf
2. Office of Environmental Health Hazard Assessment. Health effects assessment: potential neurobehavioral effects of synthetic food dyes in children. https://oehha.ca.gov/media/downloads/risk-assessment/report/fooddyesassessmentdraft082820.pdf
3. (Kobylewski and Jacobson 2012)
4. (Arnold, Lofthouse et al. 2012)
5. (Boris and Mandel 1994)
6. (McCann, Barrett et al. 2007)
7. (Rowe and Rowe 1994)
8. (Schab and Trinh 2004)
9. (Potera 2010)
10. (Peiperl, Prival et al. 1995)
11. (Dipalma 1990)
12. (Stenius and Lemola 1976)
13. (Settipane, Chafee et al. 1976)
14. (Neuman, Elian et al. 1978)
15. (Settipane, Chafee et al. 1976)
16. Food Standards Australia New Zealand. Table of food additive permissions in the US and Europe https://www.foodstandards.gov.au/consumer/additives/pages/tableoffoodadditivep5753.aspx
17. (Fernando and Clarke 2009)
18. WebMD. Canthaxanthin - Uses, Side Effects, and More. https://www.webmd.com/vitamins/ai/ingredientmono-846/canthaxanthin
19. (Chen, Yan et al. 2014, Suzuki, Miura et al. 2020)
20. (Krewski, Yokel et al. 2007)
21. (Jovanović, Jovanović et al. 2018)

Arnold, L.E., Lofthouse, N. & Hurt, E. (2012). Artificial food colors and attention-deficit/hyperactivity symptoms: conclusions to dye for. *Neurotherapeutics*, 2012 *9*(3), 599-609. https://doi.org/10.1007/s13311-012-0133-x

Boris, M. & Mandel, F. S. (1994). Foods and additives are common causes of the attention deficit hyperactive disorder in children. *Ann Allergy*, *72*(5), 462-468.

Center for Science in the Public Interest. Food dyes: a rainbow of risks. https://www.cspinet.org/sites/default/files/attachment/food-dyes-rainbow-of-risks.pdf

Chen, T., Yan, J. & Li, Y. (2014). Genotoxicity of titanium dioxide nanoparticles. *J Food Drug Anal. 22*(1):95-104. https://doi.org/10.1016/j.jfda.2014.01.008

Dipalma, J. R. (1990). "Tartrazine sensitivity." *Am Fam Physician 42*(5), 1347-1350.

Fernando, S. L. & Clarke, L. R. (2009). Salicylate intolerance: a masquerader of multiple adverse drug reactions. *BMJ Case Rep*, 2009:bcr02.2009.1602. https://doi.org/10.1136/bcr.02.2009.1602

Food Standards Australia New Zealand. Table of food additive permissions in the US and Europe. https://www.foodstandards.gov.au/consumer/additives/pages/tableoffoodadditivep5753.aspx

Jovanović, B., Jovanović, N., Cvetković, V. J., Matić, S., Stanić, S., Whitley, E. M. & Mitrović, T.L. (2018). The effects of a human food additive, titanium dioxide nanoparticles E171, on Drosophila melanogaster - a 20 generation dietary exposure experiment. *Sci Rep 8*(1),17922. https://doi.org/10.1038/s41598-018-36174-w

Kobylewski, S. & Jacobson, M. F. (2012). Toxicology of food dyes. *Int J Occup Environ Health 18*(3): 220-246. https://doi.org/10.1179/1077352512Z.00000000034

Krewski, D., Yokel, R. A., Nieboer, E., Borchelt, D., Cohen, J., Harry, J., Kacew, S., Lindsay, J., Mahfouz, A. M. & Rondeau, V. (2007). Human health risk assessment for aluminium, aluminium oxide, and aluminium hydroxide. *J Toxicol Environ Health B Crit Rev 10*(Suppl 1), 1-269. https://doi.org/10.1080/10937400701597766

McCann, D., Barrett, A., Cooper, A., Crumpler, D., Dalen, L., Grimshaw, K., Kitchin, E., Lok, K., Porteous, L., Prince, E., Sonuga-Barke, E., Warner, J. O. & Stevenson, J. (2007). Food additives and hyperactive behaviour in 3-year-old and 8/9-year-old children in the community: a randomised, double-blinded, placebo-controlled trial. *Lancet, 370*(9598):1560-1567. https://doi.org/10.1016/S0140-6736(07)61306-3

Neuman, I., Elian, R., Nahum, H., Shaked, P. & Creter D. (1978). The danger of "yellow dyes" (tartrazine) to allergic subjects. Clin Allergy. 1978 Jan;8(1):65-8. https://doi.org/10.1111/j.1365-2222.1978.tb00449.x

Office of Environmental Health Hazard Assessment. Health effects assessment: potential neurobehavioral effects of synthetic food dyes in children. https://oehha.ca.gov/media/downloads/risk-assessment/report/fooddyesassessmentdraft082820.pdf

Peiperl, M. D., Prival, M. J. & Bell, S. J. (1995). Determination of combined benzidine in FD&C Yellow No. 6 (Sunset Yellow FCF). *Food Chem Toxicol 33*(10), 829-839. https://doi.org/10.1016/0278-6915(95)00051-3

Potera, C. (2010). The artificial food dye blues. *Environ Health Perspect, 118*(10), A428. https://doi: 10.1289/ehp.118-a428

Rowe, K. S. & Rowe, K. J. (1994). Synthetic food coloring and behavior: a dose response effect in a double-blind, placebo-controlled, repeated-measures study. *J Pediatr 125*(5 Pt 1): 691-698. https://doi.org/10.1016/s0022-3476(94)70059-1

Schab, D. W. & Trinh, N. H. (2004). Do artificial food colors promote hyperactivity in children with hyperactive syndromes? A meta-analysis of double-blind placebo-controlled

trials. *J Dev Behav Pediatr 25*(6), 423-434. https://doi.org/10.1097/00004703-200412000-00007

Settipane, G. A., Chafee, F. H., Postman, I. M., Levine, M.I., Saker, J. H., Barrick, R. H., Nicholas, S. S., Schwartz, H. J., Honsinger, R. W. & Klein, D.E. (1976). Significance of tartrazine sensitivity in chronic urticaria of unknown etiology. *J Allergy Clin Immunol 57*(6), 541-546. https://doi.org/10.1016/0091-6749(76)90004-x

Stenius, B. S. & Lemola, M. (1976). Hypersensitivity to acetylsalicylic acid (ASA) and tartrazine in patients with asthma. *Clin Allergy 6*(2): 119-129. https://doi.org/10.1111/j.1365-2222.1976.tb01889.x

Suzuki, T., Miura, N., Hojo, R., Yanagiba, Y., Suda, M., Hasegawa, T., Miyagawa, M. & Wang, R. S. (2020). Genotoxicity assessment of titanium dioxide nanoparticle accumulation of 90 days in the liver of gpt delta transgenic mice. *Genes Environ 42*:10. https://doi.org/10.1186/s41021-020-00151-5

WebMD. Canthaxanthin - uses, side effects, and more. https://www.webmd.com/vitamins/ai/ingredientmono-846/canthaxanthin

Supplementary Table B. Food Additives. Synthetic and natural preservatives linked to potential harms

Synthetic Preservatives	Food Additive	Not Approved for use in the following countries	Potential links to the following harms
E201	Sodium sorbate	EU	Possible genotoxic effects
E203	Calcium sorbate	EU	Possible genotoxic effects
E211	Sodium benzoate	None	Inflammation, the development of ADHD related symptoms in children and college aged students, appetite control increasing risk for obesity, oxidative stress and allergies
E212	Potassium benzoate	None	Hives, chronic stuff nose and other allergic reactions, ADHD-related symptoms and hyperactivity in children
E214	Ethyl 4-hydroxybenzoate	France, Australia	Allergic skin reactions, not recommended for consumption by children
E215	Ethyl 4-hydroxybenzoate sodium salt	Australia	Thought to present with the same possible side effects of E214
E216	Propyl 4-hydroxybenzoate	EU, Australia	Possible allergen, not recommended for consumption by children
E217	Sodium salt of E216	Australia	Linked to possible eye damage, diarrhea, skin rash, numb mouth, intestinal cramping, throat irritation, upset stomach, headache, nausea, vomiting and rash
E225	Potassium sulfite	EU, USA	May cause allergic reactions in some sensitive persons and those intolerant to sulphites. Most studies report a prevalence of sulphite sensitivity of 3-10% among asthmatic subjects
E227	Calcium hydrogen sulphite	Australia	Should be avoided by individuals who are intolerant to sulphites. May cause severe allergic reactions
E230	Biphenyl	EU, Australia	Considered to be mildly toxic. May cause liver, central and peripheral nerve damage in excess amounts in some people. Other reported side effects include headaches and skin disorders, and it is classified as an irritant.
E231	2-hydroxybiphenyl	EU, Australia	Linked to nausea, vomiting and irritation to eyes and nose
E232	Sodium biphenyl-2-yl oxide	Australia and New Zealand	May cause nausea, vomiting and irritation to eyes and nose. Should be avoided by asthmatics or those sensitive to aspirin
E233	2-(Thiazol-4-yl)benzimidazole	EU, Australia and New Zealand	Considered likely to be carcinogenic at doses high enough to cause disturbance of the thyroid hormone balance. May cause nausea, vomiting, vertigo, diarrhoea
E236	Formic acid	EU, Australia and New Zealand	Considered to have low toxicity. May provoke the occurrence of allergic reactions and in high amounts may cause serious disorders of the gastrointestinal tract. Chronic exposure may cause kidney damage and allergic skin reactions
E237	Sodium formate	EU	May cause skin, eye and respiratory irritation
E238	Calcium formate	EU, Australia and New Zealand	Eye irritant, high levels linked to severe gastrointestinal lesions, powder inhalation considered potentially dangerous
E239	Hexamine	US, Russia, Australia or New Zealand	May cause respiratory problems such as chest tightness, coughing, wheezing and shortness of breath. May cause skin allergies such as itching or a rash. Not recommended for the consumption of children
E240	Formaldehyde	Restricted in several countries including EU, declared a "toxic substance" by Canada Environmental Protection Act	Possible carcinogenic to humans, considered an indoor air pollutant. When ingested may leads to intoxication, pale skin, headaches, weakness, shortness of breath, burning sensation in the throat, difficulty breathing and can trigger or aggravate asthma related symptoms. Eye and mucous membrane irritant. In rare cases is linked to leukemia, and nasal sinus cancer
E249	Potassium nitrite	None found	May affect breathing, may cause sneezing and coughing as well as irritate the throat and nose. High levels may impact the ability of the blood to carry oxygen leading to fatigue, headache, dizziness and a blue colour to the lips . Linked to behavioral problems. Potential carcinogen and prohibited for infants and young children.
E250	Sodium nitrite	Norway, Sweden, Canada and Germany	Associated with increased risk of heart disease, may cause arteries to harden and narrow. May also cause methemoglobinemia (a condition whereby haemoglobin loses its ability to carry oxygen) . Possible increased risk of pancreatic cancer
E924	Potassium bromate	EU, Argentina, Brazil, Canada	Possibly carcinogenic to humans
Natural Preservatives			
E284	Boric acid	China, Australia, New Zealand and Canada. Permitted to be used in caviar in the EU	Animal studies have indicated that excessive ingestion of boric acid over a prolonged period may cause adverse reproductive and developmental effects
E285	Sodium tetraborate (Borax)	EU, USA, UK	Considered harmful if swallowed and should be kept away from children. Skin irritant. Linked to respiratory problems, effects to male reproduction system, may cause vomiting, nausea, diarrhea, headaches, lightheadedness and weakness

Supplementary Table B References. Food Additives. Synthetic and natural preservatives linked to potential harms

1. (Mamur, Yüzbaşıoğlu et al. 2012)
2. (Schlatter, Würgler et al. 1992)
3. (Mamur, Yüzbaşıoğlu et al. 2010)
4. Research paper by Patel and Ramani (2017): https://www.researchgate.net/publication/339974034_In_vitro_determination_of_genotoxic_effects_of_sodium_benzoate_preservative_on_human_peripheral_blood_lymphocytes
5. (McCann, Barrett et al. 2007)
6. Article: http://www.foodcomm.org.uk/press/childrens_medicine
7. (Mancini, Paul et al. 2015)
8. Article: https://pubchem.ncbi.nlm.nih.gov/compound/7175
9. (Vally and Misso 2012)
10. Government health sheet: https://www.epa.gov/sites/default/files/2016-09/documents/biphenyl.pdf
11. (Li, Hogan et al. 2016)
12. Article: https://pubchem.ncbi.nlm.nih.gov/compound/2-Phenylphenol#section=Toxicity or National Center for Biotechnology Information (2023). PubChem Compound Summary for CID 7017, 2-Phenylphenol. Retrieved February 24, 2023 from https://pubchem.ncbi.nlm.nih.gov/compound/2-Phenylphenol
13. https://www.sciencedirect.com/topics/pharmacology-toxicology-and-pharmaceutical-science/2-hydroxybiphenyl
14. (John, Arnold et al. 2001)
15. (Agency 2012) https://www.foodstandards.gov.au/consumer/additives/pages/tableoffoodadditivep5753.aspx
16. National Library of Medicine. Formic Acid. https://webwiser.nlm.nih.gov/substance?substanceId=486&identifier=Formic%20Acid&identifierType=name&menuItemId=62&catId=83
17. (Garai, Banerjee et al. 2022)
18. (Paula Neto, Ausina et al. 2017)
19. (Sambu, Hemaram et al. 2022)
20. Center for Food Safety. Formaldehyde. https://www.cfs.gov.hk/english/programme/programme_rafs/programme_rafs_fa_02_09.html#:~:text=Ingestion%20of%20a%20small%20amount,is%20its%20cancer%20causing%20potential.
21. (Safety 2017)
22. (İnci, Zararsız et al. 2013)
23. (Songur, Ozen et al. 2010)
24. (Mortensen, Aguilar et al. 2017)
25. (Neth, Love et al. 2021)
26. (Kurokawa, Maekawa et al. 1990)
27. PubChem. Tetraborate. https://pubchem.ncbi.nlm.nih.gov/compound/Sodium-Tetraborate
28. (Bolt, Başaran et al. 2012)

Bolt, H.M., Başaran, N. & Duydu, Y. (2012), Human environmental and occupational exposures to boric acid: reconciliation with experimental reproductive toxicity data. *J Toxicol Environ Health A, 75*(8-10):508-514. https://doi.org/10.1080/15287394.2012.675301

Center for Food Safety (2017). Formaldehyde in food, the Government of Hong Kong Special Administrative Region. https://www.cfs.gov.hk/english/programme/programme_rafs/programme_rafs_fa_02_09.html#:~:text=Ingestion%20of%20a%20small%20amount,is%20its%20cancer%20causing%20potential

Eastmond, D. A. & Balakrishnan, S. (2001). Genotoxicity of pesticides. 11.4.5.1 ortho-Phenylphenol. https://www.sciencedirect.com/topics/pharmacology-toxicology-and-pharmaceutical-science/2-hydroxybiphenyl

EFSA Panel on Food Additives and Nutrient Sources Added to Food (ANS). (2017). Re-evaluation of potassium nitrite (E 249) and sodium nitrite (E 250) as food additives. *EFSA J, 15*(6): e04786. https://doi.org/10.2903/j.efsa.2017.4786

Food Standards Agency. Table of food additive permissions in the US and Europe. https://www.foodstandards.gov.au/consumer/additives/pages/tableoffoodadditivep5753.aspx

Food Commission. Banned food additives permitted in children's medicines. http://www.foodcomm.org.uk/press/childrens_medicine/

Garai, P., Banerjee, P., Sharma, P., Chatterjee, A., Bhattacharya, R. & Saha, N.C. (2022). Mechanistic insights to lactic and formic acid toxicity on benthic oligochaete worm Tubifex tubifex. *Environ Sci Pollut Res Int, 29*(58):87319-87333. https://doi.org/10.1007/s11356-022-21361-0

İnci, M., Zararsız, İ., Davarcı, M. & Görür S. (2013). Toxic effects of formaldehyde on the urinary system. *Turk J Urol 39*(1):48-52. https://doi.org/10.5152/tud.2013.010

Kurokawa, Y., Maekawa, A., Takahashi, M. & Hayashi, Y. (1990). Toxicity and carcinogenicity of potassium bromate--a new renal carcinogen. *Environ Health Perspect, 87*, 309-335. https://doi.org/10.1289/ehp.9087309

Li, Z., Hogan, K. A., Cai, C. & Rieth, S. (2016). Human health effects of biphenyl: key findings and scientific issues. *Environ Health Perspect*, *124*(6):703-712. http://dx.doi.org/10.1289/ehp.1509730.

Mamur, S., Yüzbaşıoğlu, D., Unal, F. & Aksoy, H. (2012). Genotoxicity of food preservative sodium sorbate in human lymphocytes in vitro. *Cytotechnology, 64*(5):553-62. doi: 10.1007/s10616-012-9434-5.

Mancini, F. R., Paul, D., Gauvreau, J., Volatier, J. L., Vin, K. & Hulin, M. (2015). Dietary exposure to benzoates (E210-E213), parabens (E214-E219), nitrites (E249-E250), nitrates (E251-E252), BHA (E320), BHT (E321) and aspartame (E951) in children less than 3 years old in France. *Food Addit Contam Part A Chem Anal Control Expo Risk Assess*, *32*(3):293-306. https://doi.org/10.1080/19440049.2015.1007535

McCann, D., Barrett, A., Cooper, A., Crumpler, D., Dalen, L., Grimshaw, K., Kitchin, E., Lok, K., Porteous, L., Prince, E., Sonuga-Barke, E., Warner, J. O. & Stevenson, J. (2007). Food additives and hyperactive behaviour in 3-year-old and 8/9-year-old children in the

community: a randomised, double-blinded, placebo-controlled trial. *Lancet, 370*(9598):1560-1567. doi: 10.1016/S0140-6736(07)61306-3

Neth, M. R., Love, J. S., Horowitz, B. Z., Shertz, M. D., Sahni, R., & Daya, M. R. (2021). Fatal sodium nitrite poisoning: key considerations for prehospital providers. *Prehosp Emerg Care, 25*(6):844-850. https://doi.org/10.1080/10903127.2020.1838009

Patel, D. & Ramani, R. (2017). *In vitro* determination of genotoxic effects of sodium benzoate preservative on human peripheral blood lymphocytes. *Int J Res Biosciences, 6*(3): 20-26.

Paula Neto, H. A., Ausina, P., Gomez, L. S., Leandro, J. G. B., Zancan, P. & Sola-Penna, M. (2017). Effects of food additives on immune cells as contributors to body weight gain and immune-mediated metabolic dysregulation. *Front Immunol*, *8*, 1478. https://doi.org/10.3389/fimmu.2017.01478

Sambu, S., Hemaram, U., Murugan, R. & Alsofi, A.A. (2022). Toxicological and teratogenic effect of various food additives: an updated review. *Biomed Res Int* 2022:6829409. https://doi.org/10.1155/2022/6829409

Schlatter, J., Würgler, F.E., Kränzlin, R., Maier, P., Holliger, E. & Graf, U. (1992). The potential genotoxicity of sorbates: effects on cell cycle in vitro in V79 cells and somatic mutations in Drosophila. *Food Chem Toxicol, 30(*10):843-851. https://doi.org/10.1016/0278-6915(92)90049-q

Songur, A., Ozen, O.A. & Sarsilmaz, M. (2010). The toxic effects of formaldehyde on the nervous system. *Rev Environ Contam Toxicol, 203,* 105-118. doi: 10.1007/978-1-4419-1352-4_3

St. John, M. K., Arnold, L. L., Anderson, T., Cano, M., Johansson, S.L. & Cohen, S. M. (2001). Dietary effects of ortho-phenylphenol and sodium ortho-phenylphenate on rat urothelium. *Toxicol Sci, 59*(2):346-351. https://doi.org/10.1093/toxsci/59.2.346

Vally, H. & Misso, N. L. A. (2012). Adverse reactions to the sulphite additives. *Gastroenterol Hepatol Bed Bench, 5*(1): 16-23.

Supplementary Table C. Food Additives. Synthetic and natural antioxidants linked to potential harms

Synthetic Anti-Oxidants, Stabilizers & Emulsifiers	Food Additive	Not Approved for use in the following countries	Potential links to the following harms
E310	Propl gallate	None found	Not recommended for use in children. Derived by gallic acid which may be linked to eczema, stomach complaints, hyperactivity and skin sensitivity. May act as an estrogen antagonist.
E312	Dodecyl gallate	Philippines	Skin contact may cause rashes, burning, irritation and redness. Should be avoided by asthamatics, those senstive to aspirin. Gallates are not permitted in foods for infants and small children because of their potential tendency to cause the blood disorder, methemoglobinemia
E320	Butylated hydroxyanisole (BHA)	Japan, Europe, Canada, Australia, and New Zealand. The state of California list BHA as a carcinogen	May promote tumor growth and impair blood clotting. The National Institutes of Health (NIH) report that BHA is reasonably anticipated to be carcinogenic to humans based on experimental animal studies
E321	Butylated hydroxytoluene (BHT)	Australia, Canada, New Zealand, Japan. The UK have banned the use of BHA in infant food	Concerns have been raised by WHO about its use for human consumption. As above, in high quantities may impair blood clotting and promote tumor growth.
E370	1,4-Heptonolactone	Australia and New Zealand	May cause allergies and adverse reactions
E381	Ammonium ferric citrate	EU	May irritate the skin and eyes and cause respiratory problems in some people
E388	Thiodipropionic acid	EU, Australia and New Zealand	Potential to cause eye damage, skin irritation, harmful to aquatic life
**Natural Anti-Oxidants, Stabilizers, and Emulsifiers**			
E426	Soybean hemicellulose	Australia and New Zealand. The FDA has issued warnings about certain confectionary products (jelly type sweets) containing soybean hemicellulose	Contains potentially allergenic proteins. Risk of choking in infants, children and the elderly in specific jelly-like sweets containing soybean hemicellulose

Supplementary Table C References. Food Additives. Synthetic and natural antioxidants linked to potential harms

1. (Baran, Köktürk et al. 2018)
2. (Nakagawa, Moldéus et al. 1996)
3. (van der Heijden, Janssen et al. 1986)
4. (Verhagen, Schilderman et al. 1991)
5. (Jayalakshmi and Sharma 1986)
6. (Xu, Liu et al. 2021)
7. Government Report: https://food.ec.europa.eu/system/files/2020-12/sci-com_scf_reports_25.pdf
8. (Ritz, Hahn et al. 2012)
9. (Huang, Ma et al. 2019)
10. (Wu, Geng et al. 2021)
11. Thermo Fisher safety data scientific report: https://www.fishersci.com/store/msds?partNumber=AC138750025&countryCode=US&language=en
12. National library of Medicine: https://pubchem.ncbi.nlm.nih.gov/compound/3_3_-Thiodipropionic-acid
13. https://efsa.onlinelibrary.wiley.com/doi/pdf/10.2903/j.efsa.2017.4721
14. (Taylor, Remington et al. 2015)

Baran, A., Köktürk, M., Atamanalp, M. & Ceyhun, S. B. (2018). Determination of developmental toxicity of zebrafish exposed to propyl gallate dosed lower than ADI (Acceptable Daily Intake). *Regul Toxicol Pharmacol, 94,* 16-21. https://doi.org/10.1016/j.yrtph.2017.12.027

Commission of the European Communities. (1990). Food--science and techniques. https://food.ec.europa.eu/system/files/2020-12/sci-com_scf_reports_25.pdf

EFSA panel on food additives. (2017). Re-evaluation of soybean hemicellulose (E 426) as a food additive. *EFSA J, 15*(3):4721. https://efsa.onlinelibrary.wiley.com/doi/pdf/10.2903/j.efsa.2017.4721

Huang, C., Ma, W., Luo, Q., Shi, L., Xia, Y., Lao, C., Liu, W., Zou, Y., Cheng, A., Shi, R. & Chen, Z. (2019). Iron overload resulting from the chronic oral administration of ferric citrate induces parkinsonism phenotypes in middle-aged mice. *Aging, 11*(21), 9846-9861. https://doi.org/10.18632/aging.102433

Jayalakshmi, C. P. & Sharma, J. D. (1986). Effect of butylated hydroxyanisole (BHA) and butylated hydroxytoluene (BHT) on rat erythrocytes. *Environ Res 41*(1), 235-238. https://doi.org/10.1016/s0013-9351(86)80185-2

Nakagawa, Y., Moldéus, P. & Moore, G.A. (1996). Relationship between mitochondrial dysfunction and toxicity of propyl gallate in isolated rat hepatocytes. *Toxicology 114*(2), 135-145. https://doi.org/10.1016/s0300-483x(96)03479-8

National Library of Medicine. 3,3'-Thiodipropionic acid. https://pubchem.ncbi.nlm.nih.gov/compound/3_3_-Thiodipropionic-acid

Ritz, E., Hahn, K., Ketteler, M., Kuhlmann, M. K. & Mann, J. (2012). Phosphate additives in food--a health risk. *Dtsch Arztebl Int 109*(4): 49-55. https://doi.org/10.3238/arztebl.2012.0049

Taylor, S. L., Remington, B. C., Panda, R. Goodman, R. E. & Baumert, J. L. (2015). 18 - Detection and control of soybeans as a food allergen. In: Flanagan, S. (ed), Handbook of Food Allergen Detection and Control, Woodhead Publishing Series in Food Science, Technology and Nutrition, pp. 341-366. https://doi.org/10.1533/9781782420217.3.341

van der Heijden, C. A., Janssen, P.J. & Strik, J. J. (1986). Toxicology of gallates: a review and evaluation. *Food Chem Toxicol, 24*(10-11), 1067-1070. https:doi.org/10.1016/0278-6915(86)90290-5

Verhagen, H., Schilderman, P. A. E. L., & Kleinjans, J. C. S. (1991). Butylated hydroxyanisole in perspective. *Chem Biol Interact, 80*(2), 109-134. https://doi.org/10.1016/0009-2797(91)90019-4

Wu, W., Geng, Z., Bai, H., Liu, T. & Zhang, B. (2021). Ammonium ferric citrate induced ferroptosis in non-small-cell lung carcinoma through the inhibition of GPX4-GSS/GSR-GGT axis activity. *Int J Med Sci, 18*(8):1899-1909. https://doi.org/10.7150/ijms.54860

Xu, X., Liu, A., Hu, S., Ares, I., Martínez-Larrañaga, M. R., Wang, X., Martínez, M., Anadón, A. & Martínez, M. A. (2021). Synthetic phenolic antioxidants: metabolism, hazards and mechanism of action. *Food Chem, 353,* 129488. https://doi.org/10.1016/j.foodchem.2021.129488

Supplementary Table D. Food Additives. Synthetic and natural thickening agents and emulsifiers linked to potential harms

Synthetic Thickening Agents and Emulsifiers	Food Additive	Not Approved for use in the following countries	Potential links to the following harms
E432	Polyoxyethylene-20-sorbitan monolaurate	Australia in dairy products	The acute toxicity is considered very low. People intolerant of propylene glycol should also avoid the group of 430-E436
E434	Polyoxyethylene-20-sorbitan monopalmitate	Australia and New Zealand	Public health concerns regarding two carcinogens: ethylene oxide, 1,4-dioxane as well as potential for blood brain barrier disruption, and some allergic symptoms
E459	Beta-cyclodextrin	Australia and New Zealand	May cause respiratory tract irritation. May be harmful to the skin if absorbed. May be an irritant to the eyes.
E469	Enzymatically hydrolysed carboxy methyl cellulose	Australia and New Zealand	Case report data of severe allergic reactions (although considered uncommon), may alter gut microbiota, may trigger inflammatory bowel disease, may cause inflmaation of the gut. On-going research
E474	Sucroglycerides	Australia	Can be derived from both plant and animal fat, Should be avoided by vegans. May be linked to food intolerances
E493	Sorbitan monolaurate	Australia and New Zealand	Sorbitol is associated with gastrointestinal complaints including gas and diarrhoea. Not permitted in infants and young children. May be an irritant to the skin and eyes
E494	Sorbitan monooleate	Australia and New Zealand	May be an irritant to the skin and eyes
E495	Sorbitan monopalmitate	Australia and New Zealand	Possible mild irritant if in direct contat with skin
**Natural Thickening Agents and Emulsifiers**			
E303	Potassium ascorbate	UK, USA and EU	May cause allergic skin and eye reactions in some people although carries a low risk. Evaluation of potential harms and safety currently incomplete
E350i	Sodium hydrogen DL malate	EU, Australia and New Zealand	Malic acid and its salts are considered as strongly irritant to the skin and mucosa and as a particular risk to the eyes.
E350ii	Sodium DL malate	Australia and New Zealand	Excessive consumption may cause irritation of the mouth
E356	Sodium adipate	Australia and New Zealand	May cause eye irritation, may cause respiratory irritation. Considered harmful to aquatic life
E387	Oxystearin	Australia and New Zealand	Prepared from both plant and animal origin. Should be avoided by vegans

Supplementary Table D References. Food Additives. Synthetic and natural thickening agents and emulsifiers linked to potential harms

1. (Zar, Graeber et al. 2007)
2. (Pillai, Hothi et al. 2014)
3. Science direct link: https://www.sciencedirect.com/topics/medicine-and-dentistry/propylene-glycol
4. European Medicines Agency online article: https://www.ema.europa.eu/en/documents/report/propylene-glycol-used-excipient-report-published-support-questions-answers-propylene-glycol-used_en.pdf
5. (Fowles, Banton et al. 2013)
6. (Cawley 2001)
7. (Harris, Sherman et al. 1951)
8. (Gould and Scott 2005)
9. (Juśkiewicz and Zduńczyk 2004)
10. (Miclotte, De Paepe et al. 2020)
11. (Naimi, Viennois et al. 2021)
12. (Partridge, Lloyd et al. 2019)
13. (Holder, Peters et al. 2019)
14. (Harris, Sherman et al. 1951)
15. (Bampidis, Azimonti et al. 2019) https://www.efsa.europa.eu/en/efsajournal/pub/5651
16. (Bampidis, Azimonti et al. 2019)
17. https://www.ewg.org/skindeep/ingredients/705225-potassium_sorbate
18. https://www.webmd.com/vitamins/ai/ingredientmono-1495/malic-acid
19. (Liu, Zhan et al. 2022)
20. (Hadrup, Frederiksen et al. 2022)
21. (Witkowski, Grajeta et al. 2022)

Cawley, M. J. (2001). Short-term lorazepam infusion and concern for propylene glycol toxicity: case report and review. *Pharmacotherapy 21*(9): 1140-1144. https://doi.org/10.1592/phco.21.13.1140.34611

EFSA Panel on Additives and Products or Substances used in Animal Feed (2019). Safety and efficacy of sorbitan monolaurate as a feed additive for all animal species. *EFSA J, 17*(3), e05651. https://doi.org/10.2903/j.efsa.2019.5651

Environmental Working Group. Potassium sorbate. https://www.ewg.org/skindeep/ingredients/705225-potassium_sorbate/

European Medicines Agency. Propylene glycol. https://www.ema.europa.eu/en/documents/report/propylene-glycol-used-excipient-report-published-support-questions-answers-propylene-glycol-used_en.pdf

Fowles, J. R., Banton, M. I. & Pottenger, L. H. (2013). A toxicological review of the propylene glycols. Crit Rev Toxicol. 43(4), 363-390. https://doi.org/10.3109/10408444.2013.792328.

Gould, S. & Scott, R. C. (2005). 2-Hydroxypropyl-beta-cyclodextrin (HP-beta-CD): a toxicology review. *Food Chem Toxicol 43*(10), 1451-1459. https://doi.org/10.1016/j.fct.2005.03.007

Hadrup, N., Frederiksen, M., Wedebye, E. B., Nikolov, N. G., Carøe, T. K., Sørli, J. B., Frydendall, K. B., Liguori, B., Sejbaek, C. S., Wolkoff, P., Flachs, E. M., Schlünssen, V., Meyer, H. W., Clausen, P. A. & Hougaard, K. S. (2022). Asthma-inducing potential of 28 substances in spray cleaning products-Assessed by quantitative structure activity relationship (QSAR) testing and literature review. *J Appl Toxicol 42*(1):130-153. https://doi.org/10.1002/jat.4215

Harris, R. S., Sherman, H. & Jeter. W. W. (1951). Nutritional and pathological effects of sorbitan monolaurate, polyoxyethylene sorbitan monolaurate, polyoxyethylene monolaurate, and polyoxyethylene monostearate when fed to rats. *Arch Biochem Biophys 34*(2), 249-258. https://doi.org/10.1016/0003-9861(51)90002-1

Holder, M. K., Peters, N. V., Whylings, J., Fields, C. T., Gewirtz, A. T., Chassaing, B. & de Vries, G. J. (2019). Dietary emulsifiers consumption alters anxiety-like and social-related behaviors in mice in a sex-dependent manner. *Sci Rep, 9*(1):172. doi: 10.1038/s41598-018-36890-3.

Juśkiewicz, J. & Zduńczyk, Z. (2004). Effects of cellulose, carboxymethylcellulose and inulin fed to rats as single supplements or in combinations on their caecal parameters. *Comp Biochem Physiol A Mol Integr Physiol, 139*(4), 513-519. https://doi.org/10.1016/j.cbpb.2004.10.015

Liu, C., Zhan, S., Tian, Z., Li, N., Li, T., Wu, D., Zeng, Z. & Zhuang, X. (2022). Food additives associated with gut microbiota alterations in inflammatory bowel disease: friends or enemies? *Nutrients, 14*(15):3049. https://doi.org/10.3390/nu14153049

Miclotte, L., De Paepe, K., Rymenans, L., Callewaert, C., Raes, J., Rajkovic, A., Van Camp, J. & Van de Wiele, T. (2020). Dietary emulsifiers alter composition and activity of the human gut microbiota *in vitro*, irrespective of chemical or natural emulsifier origin. *Front Microbiol, 11*:577474. https://doi.org/10.3389/fmicb.2020.577474

Naimi, S., Viennois, E., Gewirtz, A. T., Chassaing, B. (2021). Direct impact of commonly used dietary emulsifiers on human gut microbiota. *Microbiome, 9*(1):66. https://doi.org/10.1186/s40168-020-00996-6

Partridge, D., Lloyd, K. A., Rhodes, J. M., Walker, A. W., Johnstone, A. M. & Campbell, B. J. (2019). Food additives: Assessing the impact of exposure to permitted emulsifiers on bowel and metabolic health - introducing the FADiets study. *Nutr Bull, 44*(4):329-349. https://doi.org/10.1111/nbu.12408

Pillai, U., Hothi, J. C. & Bhat, Z. Y. (2014). Severe propylene glycol toxicity secondary to use of anti-epileptics. *Am J Ther, 21*(4):e106-9. https://doi.org/10.1097/MJT.0b013e31824c407d

Witkowski, M., Grajeta, H. & Gomułka, K. (2022). Hypersensitivity reactions to food additives-preservatives, antioxidants, flavor enhancers. *Int J Environ Res Public Health, 19*(18):11493. https://doi.org/10.3390/ijerph191811493

Zar, T., Graeber, C., Perazella, M. A. (2007), Recognition, treatment, and prevention of propylene glycol toxicity. *Semin Dial, 20*(3):217-219.

https://doi.org/10.1111/j.1525-139X.2007.00280.x

Supplementary Table E. Food Additives. Synthetic sweeteners and natural flavor enhancers linked to potential harms

Natural Flavor Enhancers	Food Additive	Not Approved for use in the following countries	Potential links to the following harms
E626	Guanylic acid (and its salts which include sodium guanylate (E627), di-potassium guanylate (E628) and calcium guanylate (E629)	Australia and New Zealand	Should be avoided by those suffering with asthma or gout. Frequently used in combination with MSG. GMO
E627	Sodium guanylate	Australia and New Zealand	It is not permitted for use in babies under 12 weeks of age. Guanylic acid and guanylate should be avoided by asthma sufferers and those with gout. As a food additive, guanylic acid produced by sardines would not be suitable for vegans or vegetarians
E628	Di-potassium guanylate	Australia and New Zealand	As above
E629	Calcium guanylate	Australia and New Zealand	As above
E630	Inosinic acid	Australia and New Zealand	Asthmatic's and those with gout should avoid the consumption of inosinates. They should not be in included in products for babies under 12 weeks of age
E634	Calcium ribonucleotides	Australia and New Zealand	Guanylates and inosinates should not be used in products for babies under 12 weeks of age
**Synthetic Sweeteners**	**Food Additive**		
E952	Cyclamates	USA	Has been banned for human consumption since 1970 by the U.S. Food and Drug Administration due health concerns ans potential links to cancer

Supplmentary Table E References. Food Additives. Synthetic sweeteners and natural flavor enhancers linked to potential harms

1. (Kallscheuer 2018)
2. (Baines and Brown 2016) - Science Direct: https://www.sciencedirect.com/topics/agricultural-and-biological-sciences/guanosine-monophosphate
3. (Kurihara 2015)
4. (Gregson and Simmonds 1971)
5. (Maiuolo, Oppedisano et al. 2016)
6. (Mahbub, Yamaguchi et al. 2017)
7. (1999)(1999). "Cyclamates." IARC Monogr Eval Carcinog Risks Hum **73**: 195-222.

Baines, D. & Brown, M. (2016). Flavor enhancers: characteristics and uses. In: Caballero, B., Finglas. P. M. & Toldrá, F. (eds). *Encyclopedia of Food and Health.* Oxford, Academic Press**:** pp. 716-723. https://www.sciencedirect.com/referencework/9780123849533/encyclopedia-of-food-and-health#browse-content

Gregson, R. A. & M. B. Simmonds (1971). Qualitative gustatory characteristics of disodium-5'-guanylate *Br J Psychol 62*(1), 81-88. https://doi.org/10.1111/j.2044-8295.1971.tb02014.x

Kallscheuer, N. (2018). Engineered microorganisms for the production of food additives approved by the European Union-a systematic analysis. *Front Microbiol 9*, 1746. https://doi.org/10.3389/fmicb.2018.01746

Kurihara, K. (2015). Umami, the fifth basic taste: history of studies on receptor mechanisms and role as a food flavor. *Biomed Res Int, 2015*:189402. https://doi.org/10.1155/2015/189402

Mahbub, M. H., Yamaguchi, N., Takahashi, H., Hase, R., Amano, H., Kobayashi-Miura, M., Kanda, H., Fujita, Y., Yamamoto, H., Yamamoto, M., Kikuchi, S., Ikeda, A., Kageyama, N., Nakamura, M., Ishimaru, Y., Sunagawa, H. **&** Tanabe, T. (2017). Alteration in plasma free amino acid levels and its association with gout. *Environ Health Prev Med, 22*(1):7. https://doi.org/10.1186/s12199-017-0609-8

Maiuolo, J., Oppedisano, F., Gratteri, S., Muscoli, C. & Mollace, V. (2016). Regulation of uric acid metabolism and excretion. *Int J Cardiol, 213*:8-14. https://doi.org/10.1016/j.ijcard.2015.08.109.

Schmidt, R. & Lens, L. L. (2014) Adjuvants targeting the DNA sensing pathways --cyclic diGMP and other cyclic dinucleotides. In: Ishii K. J. & Tang, C. K. (eds). *Biological DNA Sensor: the Impact of Nucleic Acids on Disease and Vaccinology.* Oxford, Academic Press. https://www.sciencedirect.com/book/9780124047327/biological-dna-sensor#book-info

Supplementary Table F. Criteria to escape Tier III

Criterion	Comment
No added trans-fats or artificially hydrogenated or partially hydrogenated vegetable oils or margarine	
No more than 2 g of added fructose per serving, whole fruit excluded	
(Target of) no more than 4 g of added glucose per serving	• AHA: 6 tsp for women, 9 tsp for men = 25 – 37.5g of added sugar/day<br>• WHO (rescinded guidelines): 5% of daily calories (2000) = 25g of added sugar/day<br>• AAP: 6 tsp = 25g of added sugar/day<br>• USDA 2020-2025 guidelines: 6% of daily calories (2000) = 30g of added sugar/day
No additives with health risks<br>No synthetic emulsifiers in fermented milk products	See **Supplementary Tables 1-4**
Less than 3:1 ratio of omega-6 to omega-3 fatty acids per serving<br>No more than 5g of omega-6 fatty acids per serving	
Less than 2:1 ratio of kilocalories to milligrams of sodium	With a recommended range of 1,600 to 2,400 daily calorie consumption for the majority of consumers, the recommendation of 2,300 milligrams of daily sodium would be a ratio of approximately 1:1. Given the variation in KDD products, the proposed ratio of 2:1 allows for improved sodium targets in the product range
No more than 0.02 ppm of lead<br>No more than 0.03 ppm of cadmium<br>No more than 0.01 ppm of arsenic<br>No more than 0.05 ppm of mercury<br>No more than 0.04 ppm of glyphosate	10% of WHO threshold for milk<br>10% of WHO threshold for cocoa<br>10% of WHO threshold for rice<br>10% of WHO threshold for fish<br>4% of 1 mg of glyphosate per kg of bodyweight per day. See doi:10.1289/EHP6990
Below minimum thresholds of toxic substances:<br>• agrochemicals and atrazine in products<br>• obesogens bisphenol A + S (BPA, BPS), phthalates, per- and polyfluoroalkyl substances (PFAS) in packaging<br>• antibiotics<br>• BST	

Supplementary Table F References. Food Additives. Criteria to escape Tier III

No additives with health risks – please see Word documents titled Supplementary Tables A-E

No synthetic emulsifiers in fermented milk products

1. (Chassaing, Compher et al. 2022)
2. (Partridge, Lloyd et al. 2019)
3. (Rousta, Oka et al. 2021)
4. (Chassaing, Koren et al. 2015)
5. (Chassaing, Van de Wiele et al. 2017)
6. (Bancil, Sandall et al. 2021)
7. (Laster, Bonnes et al. 2019)
8. (Cox, Sandall et al. 2021)
9. (Halmos, Mack et al. 2019)
10. (Roberts, Rushworth et al. 2013)

References

Bancil, A. S., Sandall, A. M., Rossi, M., Chassaing, B., Lindsay, J. O. & Whelan, K. (2021). Food additive emulsifiers and their impact on gut microbiome, permeability, and inflammation: mechanistic insights in inflammatory bowel disease. *J Crohns Colitis, 15*(6), 1068-1079. https://doi.org/10.1093/ecco-jcc/jjaa254

Chassaing, B., Compher, C., Bonhomme, B., Liu, Q., Tian, Y., Walters, W., Nessel, L., Delaroque, C., Hao, F., Gershuni, V., Chau, L., Ni, J., Bewtra, M., Albenberg, L., Bretin, A., McKeever, L, Ley, R. E., Patterson, A. D., Wu, G. D., Gewirtz, A. T. & Lewis, J. D. (2022). Randomized controlled-feeding study of dietary emulsifier carboxymethylcellulose reveals detrimental impacts on the gut microbiota and metabolome. *Gastroenterology, 162*(3):743-756. https://doi.org/10.1053/j.gastro.2021.11.006

Chassaing, B., Koren, O., Goodrich, J. K., Poole, A. C., Srinivasan, S., Ley, R. E. & Gewirtz A. T. (2015). Dietary emulsifiers impact the mouse gut microbiota promoting colitis and metabolic syndrome. *Nature, 519*(7541), 92-96. https://doi.org/10.1038/nature14232

Chassaing, B., Van de Wiele, T., De Bodt, J., Marzorati, M. & Gewirtz, A. T. (2017). Dietary emulsifiers directly alter human microbiota composition and gene expression ex vivo potentiating intestinal inflammation. *Gut, 66*(8):1414-1427. https://doi.org/10.1136/gutjnl-2016-313099

Cox, S., Sandall, A., Smith, L., Rossi, M. & Whelan, K. (2021). Food additive emulsifiers: a review of their role in foods, legislation and classifications, presence in food supply, dietary exposure, and safety assessment. *Nutr Rev, 79*(6):726-741. https://doi.org/10.1093/nutrit/nuaa038

Halmos, E. P., Mack, A. & Gibson, P. R. (2019). Review article: emulsifiers in the food supply and implications for gastrointestinal disease. *Aliment Pharmacol Ther, 49*(1), 41-50. https://doi.org10.1111/apt.15045

Laster, J., Bonnes, S. L. & Rocha, J . (2019). Increased use of emulsifiers in processed foods and the links to obesity." *Curr Gastroenterol Rep, 21*(11), 61. https://doi.org/10.1007/s11894-019-0723-4

Partridge, D., Lloyd, K. A., Rhodes, J. M., Walker, A. W., Johnstone, A. M. & Campbell, B. J. (2019). Food additives: Assessing the impact of exposure to permitted emulsifiers on bowel and metabolic health - introducing the FADiets study. *Nutr Bull, 44*(4):329-349. https://doi.org/10.1111/nbu.12408

Roberts, C. L., Rushworth, S. L., Richman, E. & Rhodes, J. M. (2013). Hypothesis: Increased consumption of emulsifiers as an explanation for the rising incidence of Crohn's disease. *J Crohns Colitis* 7(4), 338-341. https://doi.org/10.1016/j.crohns.2013.01.004

Rousta, E., Oka, A., Liu, B., Herzog, J., Bhatt, A. P., Wang, J., Habibi Najafi M. B., Sartor, R. B. (2021). The emulsifier carboxymethylcellulose induces more aggressive colitis in humanized mice with inflammatory bowel disease microbiota than polysorbate-80. *Nutrients, 13*(10):3565. https://doi.org/10.3390/nu13103565

Supplementary Table F. Food Additives. Criteria to escape Tier III

Added Fructose: No more than 2 g of added fructose per serving, whole fruit excluded

1. (Herman and Birnbaum 2021)
2. (Shi, Liu et al. 2021)
3. (Smith, Dyson et al. 2022)
4. (Merino, Fernández-Díaz et al. 2019, Muriel, López-Sánchez et al. 2021)
5. (Hannou, Haslam et al. 2018)
6. (Febbraio and Karin 2021)
7. (Spagnuolo, Iossa et al. 2020)
8. (Jung, Bae et al. 2022)
9. (Jang, Hui et al. 2018)
10. (Turck, Bohn et al. 2022)

EFSA Panel on Nutrition, Novel Foods and Food Allergens (NDA). Tolerable upper intake level for dietary sugars." *EFSA J, 20*(2): e07074. https://doi.org/10.2903/j.efsa.2022.7074

Febbraio, M. A. & M. Karin, M. (2021). "Sweet death": Fructose as a metabolic toxin that targets the gut-liver axis. *Cell Metab, 33*(12), 2316-2328. https://doi.org/10.1016/j.cmet.2021.09.004

Hannou, S. A., Haslam, D. E., McKeown, N. M., Herman, M. A. (2018). Fructose metabolism and metabolic disease. *J Clin Invest, 128*(2):545-555. https://doi.org/10.1172/JCI96702

Herman, M. A. & Birnbaum, M. J. (2021). Molecular aspects of fructose metabolism and metabolic disease. *Cell Metab, 33*(12), 2329-2354. https://doi.org/10.1016/j.cmet.2021.09.010

Jang, C., Hui, S., Lu, W., Cowan, A. J., Morscher, R. J., Lee, G., Liu, W., Tesz, G. J., Birnbaum, M. J. & Rabinowitz, J. D. (2018). The small intestine converts dietary fructose into glucose and organic acids. *Cell Metab, 27*(2):351-361.e3. https://doi.org/10.1016/j.cmet.2017.12.016

Jung, S., Bae, H., Song, W. S. & Jang, C. (2022). Dietary fructose and fructose-induced pathologies. *Ann Rev Nutr 42*, 45-66. https://doi.org/10.1146/annurev-nutr-062220-025831

Merino, B., Fernandez-Diaz, C. M., Cózar-Castellano, I. & Perdomo, G. (2019). "Intestinal fructose and glucose metabolism in health and disease. *Nutrients, 12*(1), 94. https://doi.org/10.3390/nu12010094

Muriel, P., Lopez-Sanchez, P. & Ramos-Tovar, E. (2021). Fructose and the Liver. *Int J Mol Sci 22*(13), 6969. https://doi.org/10.3390/ijms22136969

Shi, Y. N., Liu, Y. J., Xie, Z., Zhang, W. J. (2021). Fructose and metabolic diseases: too much to be good. *Chin Med J, 134*(11), 1276-1285. https://doi.org/10.1097/CM9.0000000000001545

Smith, E. V.L., Dyson, R. M., Weth, F. R., Berry, M. J. & Gray, C. (2022) Maternal fructose intake, programmed mitochondrial function and predisposition to adult disease. *Int J Mol Sci, 23(*20):12215. https://doi.org/10.3390/ijms232012215

Spagnuolo, M. S., Iossa, S. & Cigliano, L. (2020). Sweet but bitter: focus on fructose impact on brain function in rodent models." Nutrients **13**(1), 1. https://doi.org/10.3390/nu13010001

Supplementary Table F. Food Additives. Criteria to escape Tier III

(Target of) no more than 4 g of added glucose per serving

References

1. (Johnson, Appel et al. 2009)
2. (Vos, Kaar et al. 2017)
3. (Pan and Hu 2011)
4. (Malik and Hu 2022)
5. (Hu 2013)
6. (Malik and Hu 2019)
7. (Calcaterra, Cena et al. 2023)
8. (Mozaffarian 2016)
9. (Stanhope 2016)
10. (Rupérez, Mesana et al. 2019)

References

Calcaterra, V., Cena, H., Magenes, V. C., Vincenti, A., Comola, G., Beretta, A., Di Napoli, I. & Zuccotti, G. (2023). Sugar-sweetened beverages and metabolic risk in children and adolescents with obesity: a narrative review. *Nutrients, 15*(3), 702. https://doi.org/10.3390/nu15030702

Hu, F. B. (2013). Resolved: there is sufficient scientific evidence that decreasing sugar-sweetened beverage consumption will reduce the prevalence of obesity and obesity-related diseases. *Obes Rev, 14*(8), 606-619. https://doi.org/10.1111/obr.12040

Johnson, R. K., Appel, L. J., Brands, M., Howard, B. V., Lefevre, M., Lustig, R. H., Sacks, F., Steffen, L. M. & Wylie-Rosett, J. (2009). Dietary sugars intake and cardiovascular health: a scientific statement from the American Heart Association. *Circulation, 120*(11), 1011-1020. https://doi.org/10.1161/CIRCULATIONAHA.109.192627

Malik, V. S. & Hu, F. B. (2019). Sugar-sweetened beverages and cardiometabolic health: an update of the evidence. *Nutrients 11*(8), 1840. https://doi.org/10.3390/nu11081840

Malik, V. S. & Hu, F. B. (2022). The role of sugar-sweetened beverages in the global epidemics of obesity and chronic diseases. *Nat Rev Endocrinol, 18*(4), 205-218. https://doi.org/10.1038/s41574-021-00627-6

Mozaffarian, D. (2016). Dietary and policy priorities for cardiovascular disease, diabetes, and obesity: a comprehensive review. *Circulation 133*(2), 187-225. https://doi.org/10.1161/CIRCULATIONAHA.115.018585

Pan, A. & Hu, F. B. (2011). Effects of carbohydrates on satiety: differences between liquid and solid food. *Curr Opin Clin Nutr Metab Care, 14*(4), 385-390. https://doi.org/10.1097/MCO.0b013e328346df36

Rupérez, A. I., Mesana, M. I. & Moreno, L. A. (2019). Dietary sugars, metabolic effects and child health. *Curr Opin Clin Nutr Metab Care, 22*(3), 206-216. https://doi.org/10.1097/MCO.0000000000000553

Stanhope, K. L. (2016). Sugar consumption, metabolic disease and obesity: the state of the controversy. *Crit Rev Clin Lab Sci, 53*(1), 52-67. https://doi.org/10.3109/10408363.2015.1084990

Vos MB, Kaar JL, Welsh JA, Van Horn LV, Feig DI, Anderson CAM, Patel MJ, Cruz Munos J, Krebs NF, Xanthakos SA, Johnson RK. (2017). "Added sugars and cardiovascular disease risk in children: a scientific statement from the American Heart Association. *Circulation, 135(*19), e1017-e1034. https://doi.org/10.1161/CIR.0000000000000439

Supplementary Table F References. Food Additives. Criteria to escape Tier III

Heavy metals: *No more than 0.02 ppm of lead (refs 1-10); No more than 0.03 ppm of cadmium; No more than 0.01 ppm of arsenic; No more than 0.05 ppm of mercury* and: *No more than 0.04 ppm of glyphosate*

Lead References: 1-10

1. FDA government online article: https://www.fda.gov/food/environmental-contaminants-food/lead-food-foodwares-and-dietary-supplements
2. FDA government online article: https://www.fda.gov/food/environmental-contaminants-food/what-you-can-do-limit-exposure-arsenic-and-lead-juices
3. (Vasconcelos Neto, Silva et al. 2019)

4. (Cabrera, Gallego et al. 2020)
5. (Kumar, Kumar et al. 2020)
6. European Commission online article: https://food.ec.europa.eu/safety/chemical-safety/contaminants/catalogue/lead_en#:~:text=Cereal%20products%20and%20grains%2C%20vegetables,of%20the%20general%20adult%20population.
7. (Union 2006)
8. Environmental Defense Fund online article: https://www.edf.org/health/lead-food-hidden-health-threat
9. (Ciobanu, Slencu et al. 2012)
10. (Yao, Shao et al. 2022)

Cadmium: Refs 11-20

11. (Kim et al., 2019)
12. (Awata, Linder et al. 2017)
13. (Abt, Fong Sam et al. 2018)
14. (Alves Peixoto, Oliveira et al. 2018)
15. (Abt and Robin 2020)
16. (Genchi, Sinicropi et al. 2020)
17. (Kumar and Sharma 2019)
18. (Huang, He et al. 2017)
19. FDA Government online article: https://www.fda.gov/science-research/peer-review-scientific-information-and-assessments/external-peer-review-fdas-draft-toxicological-reference-value-cadmium
20. FDA online article: https://www.fda.gov/food/conversations-experts-food-topics/what-fda-doing-protect-consumers-toxic-metals-foods

Arsenic: Refs 21-30

21. FDA government online article: https://www.fda.gov/food/environmental-contaminants-food/arsenic-food-and-dietary-supplements
22. FDA guide to reducing inorganic arsenic in apple juice: https://www.fda.gov/media/86110/download
23. (Jomova, Jenisova et al. 2011)
24. (Hughes 2002)
25. (Medina-Pizzali, Robles et al. 2018)
26. (Brandon, Janssen et al. 2014)
27. (Mania, Rebeniak et al. 2015)
28. (Rahman, Granberg et al. 2017)
29. (Rehman, Khan et al. 2021)
30. European Commission Food Safety Government online article: https://food.ec.europa.eu/safety/chemical-safety/contaminants/catalogue/arsenic_en

Mercury: refs 31-40

31. European Commission for Food Safety online article: (EFSA Dietetic Products and Allergies 2014)
32. European Commission setting maximum levels for certain contaminants in foodstuffs: http://data.europa.eu/eli/reg/2006/1881/oj
33. (Padmakumar, Premkala Raveendran et al. 2019)

34. (Collado-López, Betanzos-Robledo et al. 2022)
35. (Bernhoft 2012)
36. (Oken and Bellinger 2008)
37. (Deroma, Parpinel et al. 2013)
38. (Castoldi, Johansson et al. 2008)
39. (Mendola, Selevan et al. 2002)
40. (Bjørklund, Chirumbolo et al. 2019)

Glyphosate: refs 41-45

41. (Myers, Antoniou et al. 2016)
42. (Milesi, Lorenz et al. 2021)
43. (Soares, Silva et al. 2021)
44. (Peillex and Pelletier 2020)
45. FDA government online article: https://www.fda.gov/food/pesticides/questions-and-answers-glyphosate

References

Abt, E., Sam, J. F., Gray, P. & Robin, L. P. (2018). Cadmium and lead in cocoa powder and chocolate products in the US market. *Food Addit Contam Part B Surveill, 11*(2), 92-102. https://doi.org/10.1080/19393210.2017.1420700

Abt, E. & Robin, L. P. (2020). Perspective on cadmium and lead in cocoa and chocolate. *J Agric Food Chem 68(*46), 13008-13015. https://doi.org/10.1021/acs.jafc.9b08295

Alves Peixoto, R. R., Oliveira, A. & Cadore, S. (2018). Risk assessment of cadmium and chromium from chocolate powder. *Food Addit Contam Part B Surveill 11*(4), 256-263. https://doi.org/10.1080/19393210.2018.1499676

Awata, H., Linder, S., Mitchell, L. E. & Delclos, G. L. (2017). Association of dietary intake and biomarker levels of arsenic, cadmium, lead, and mercury among Asian populations in the United States: NHANES 2011-2012. *Environ Health Perspect 125*(3), 314-323. https://dx.doi.org/10.1289/EHP28

Bernhoft, R. A. (2012). Mercury toxicity and treatment: a review of the literature. *J Environ Public Health 2012,* 460508. https://doi.org/10.1155/2012/460508

Bjørklund, G., Chirumbolo, S., Dadar, M., Pivina, L., Lindh, U., Butnariu, M. & Aaseth, J. (2019). Mercury exposure and its effects on fertility and pregnancy outcome. *Basic Clin Pharmacol Toxicol, 125*(4), 317-327. https://doi.org/10.1111/bcpt.13264

Brandon, E. F. A., Janssen, P. J. C. M. & de Wit Bos, L. (2014). Arsenic: bioaccessibility from seaweed and rice, dietary exposure calculations and risk assessment. *Food Addit Contam Part A Chem Anal Control Expo Risk Assess, 31*(12): 1993-2003. https://doi.org/10.1080/19440049.2014.974687

Cabrera, C., Gallego, C. Lopez, M. C., Lorenzo, M. L. & Lillo, E. (2020). Determination of levels of lead contamination in food and feed crops. *J AOAC Int, 77*(5): 1249-1252.

Castoldi, A. F., Johansson, C., Onishchenko, N., Coccini, T., Roda, E., Vahter, M., Ceccatelli, S. & Manzo, L. (2008). Human developmental neurotoxicity of methylmercury: impact of variables and risk modifiers. *Regul Toxicol Pharmacol, 51*(2):201-214. https://doi.org/10.1016/j.yrtph.2008.01.016

Ciobanu, C., Slencu, B. G. & Cuciureanu, R. (2012). Estimation of dietary intake of cadmium and lead through food consumption. *Rev Med Chir Soc Med Nat Iasi, 116(*2): 617-623.

Collado-López, S., Betanzos-Robledo, L., Téllez-Rojo, M. M., Lamadrid-Figueroa, H., Reyes, M., Ríos, C. & Cantoral, A. (2022). Heavy metals in unprocessed or minimally processed foods consumed by humans worldwide: a scoping review. I*nt J Environ Res Public Health, 19(*14):8651. https://doi.org/10.3390/ijerph19148651
Deroma, L., Parpinel, M., Tognin, V., Channoufi, L., Tratnik, J., Horvat, M., Valent, F. & Barbone, F. (2013). Neuropsychological assessment at school-age and prenatal low-level exposure to mercury through fish consumption in an Italian birth cohort living near a contaminated site. *Int J Hyg Environ Health, 216(*4):486-493. https://doi.org/10.1016/j.ijheh.2013.02.004

Environmental Defense Fund. Lead in food. https://www.edf.org/health/lead-food-hidden-health-threat

European Commission. Commission Regulation (EC) No 1881/2006 of 19 December 2006 setting maximum levels for certain contaminants in foodstuffs (Text with EEA relevance) https://eur-lex.europa.eu/eli/reg/2006/1881/oj

EFSA. Arsenic in food. https://food.ec.europa.eu/safety/chemical-safety/contaminants/catalogue/arsenic_en

EFSA: Lead in food. https://food.ec.europa.eu/safety/chemical-safety/contaminants/catalogue/lead_en#:~:text=Cereal%20products%20and%20grains%2C%20vegetables,of%20the%20general%20adult%20population

EFSA Dietetic Products, Nutrition, and Allergies. (2014). Scientific Opinion on health benefits of seafood (fish and shellfish) consumption in relation to health risks associated with exposure to methylmercury. *EFSA J 12*(7), 3761. https://doi.org/10.2903/j.efsa.2014.3761

Food and Drug Administration. Questions and answers on glyphosate. https://www.fda.gov/food/pesticides/questions-and-answers-glyphosate

Food and Drug Administration. External peer review of FDA's draft toxicological reference value for cadmium. https://www.fda.gov/science-research/peer-review-scientific-information-and-assessments/external-peer-review-fdas-draft-toxicological-reference-value-cadmium

Genchi, G., Sinicropi, M.S., Lauria, G., Carocci, A. & Catalano, A. (2020). The effects of cadmium toxicity. *Int J Environ Res Public Health, 17*(11), 3782. https://doi.org/10.3390/ijerph17113782

Huang, Y., He, C., Shen, C., Guo, J., Mubeen, S., Yuan, J. & Yang, Z. (2017). Toxicity of cadmium and its health risks from leafy vegetable consumption. *Food Funct, 8*(4), 1373-1401. https://doi.org/10.1039/c6fo01580h

Hughes, M. F. (2002). Arsenic toxicity and potential mechanisms of action. *Toxicol Lett 133*(1), 1-16. https://doi.org/10.1016/s0378-4274(02)00084-x

Jomova, K., Jenisova, Z., Feszterova, M., Baros, S., Liska, J., Hudecova, D., Rhodes, C. J., & Valko, M. (2011). Arsenic: toxicity, oxidative stress and human disease. *J Appl Toxicol, 31*(2), 95-107. https://doi.org/10.1002/jat.1649

Kumar, A., Kumar, A., Cabral-Pinto, M. M. S., Chaturvedi, A. K., Shabnam, A. A., Subrahmanyam, G., Mondal, R., Gupta, D. K., Malyan, S. K., Kumar, S. S., Khan S. A. & Yadav, K. K. (2020). Lead toxicity: health hazards, influence on food chain, and sustainable remediation approaches. *Int J Environ Res Public Health, 17*(7):2179. https://doi.org/10.3390/ijerph17072179

Kumar, S. & Sharma, A. (2019). Cadmium toxicity: effects on human reproduction and fertility. *Rev Environ Health 34*(4), 327-338. https://doi.org/10.1515/reveh-2019-0016

Mania, M., Rebeniak, M., Szynal, T., Wojciechowska-Mazurek, M., Starska, K., Ledzion, E. & Postupolski, J. (2015). Total and inorganic arsenic in fish, seafood and seaweeds--exposure assessment. *Rocz Panstw Zakl Hig, 66*(3):203-210.

Medina-Pizzali, M., Robles, P., Mendoza, M. & Torres, C. (2018). Ingesta de arsénico: el impacto en la alimentación y la salud humana [Arsenic Intake: Impact in Human Nutrition and Health]. *Rev Peru Med Exp Salud Publica, 35(*1):93-102.

Mendola, P., Selevan, S. G.Gutter, S. & Rice, D. (2002). Environmental factors associated with a spectrum of neurodevelopmental deficits. *Ment Retard Dev Disabil Res Rev 8*(3), 188-197. https://doi.org/10.1002/mrdd.10033

Milesi, M. M., Lorenz, V., Durando, M., Rossetti, M. F. & Varayoud, J. Glyphosate herbicide: reproductive outcomes and multigenerational effects. *Front Endocrinol, 12,* 672532. https://doi.org/10.3389/fendo.2021.672532

Myers, J.P., Antoniou, M. N., Blumberg, B., Carroll, L., Colborn, T., Everett, L. G., Hansen, M., Landrigan, P. J., Lanphear, B. P., Mesnage, R., Vandenberg, L. N., Vom Saal, F. S., Welshons, W. V. & Benbrook, C. M. (2016). Concerns over use of glyphosate-based herbicides and risks associated with exposures: a consensus statement. *Environ Health, 15*, 19. https://doi.org/10.1186/s12940-016-0117-0

Oken, E. & Bellinger, D. C. (2008). Fish consumption, methylmercury and child neurodevelopment. *Curr Opin Pediatr 20*(2), 178-183. https://doi.org/10.1097/MOP.0b013e3282f5614c

Padmakumar, V., Premkala Raveendran, K., Abdulla, A. M., Ganapathy, S., Sainudeen, S., Nasim, V. S. & Vedam, V. (2019). Levels of mercury in fish-eating children, with and without amalgam restoration. *J Pharm Bioallied Sci, 11*(Suppl 2), S397-S401. https://doi.org/10.4103/JPBS.JPBS_44_19

Peillex, C. & Pelletier, M. (2020). The impact and toxicity of glyphosate and glyphosate-based herbicides on health and immunity. *J Immunotoxicol 17*(1), 163-174. https://doi.org/10.1080/1547691X.2020.1804492

Rahman, A., Granberg, C. & Persson, L. A. (2017). Early life arsenic exposure, infant and child growth, and morbidity: a systematic review. *Arch Toxicol 91*(11), 3459-3467. https://doi.org/10.1007/s00204-017-2061-3

Rehman, M. U., Khan, R., Khan, A., Qamar, W., Arafah, A., Ahmad, A., Ahmad, A., Akhter, R., Rinklebe, J. & Ahmad, P. (2021). Fate of arsenic in living systems: Implications for sustainable and safe food chains. *J Hazard Mater, 417*:126050. https://doi.org/10.1016/j.jhazmat.2021.126050

Soares, D., Silva, L., Duarte, S., Pena, A. & Pereira, A. (2021). Glyphosate use, toxicity and occurrence in food. *Foods, 10*(11):2785. https://doi.org/10.3390/foods10112785

Vasconcelos Neto, M.C., Silva, T. B. C., Araújo, V. E. & Souza, S. V. C. (2019). Lead contamination in food consumed and produced in Brazil: systematic review and meta-analysis. *Food Res Int, 126*:108671. https://doi.org/10.1016/j.foodres.2019.108671

Yao, M., Shao, X., Wei, Y., Zhang, X., Wang, H. & Xu, F. (2022). Dietary fiber ameliorates lead-induced gut microbiota disturbance and alleviates neuroinflammation. *J Sci Food Agric, 102*(15):6795-6803. https://doi.org/10.1002/jsfa.12074

Supplementary Table F References. Food Additives. Criteria to escape Tier III

Less than 3:1 ratio of omega-6 to omega-3 fatty acids per serving (refs 1-10) and *No more than 5g of omega-6 fatty acids per serving (11-20)*

1. (Simopoulos 2002)
2. (Simopoulos 2008)
3. (Simopoulos 2006)
4. (Simopoulos 2010)
5. (Elbandy 2022)
6. (Elbandy 2022)
7. (Gómez Candela, Bermejo López et al. 2011)
8. (Simopoulos 2016)
9. (Van Name, Savoye et al. 2020)
10. (Jang and Park 2020)
11. (Blasbalg, Hibbeln et al. 2011)
12. (Ramsden, Zamora et al. 2013)
13. (Román, Jackson et al. 2019)
14. (Selmin, Papoutsis et al. 2021)
15. (Rousseau 2021)
16. (Gow and Hibbeln 2014)
17. (Hallahan, Ryan et al. 2016)
18. (Gow and Hibbeln 2014)
19. (Hibbeln and Gow 2014)
20. (Lewis, Hibbeln et al. 2011)

References:

Blasbalg, T. L., Hibbeln, J. R., Ramsden, C. E., Majchrzak, S.F., Rawlings, R. R. (2011). Changes in consumption of omega-3 and omega-6 fatty acids in the United States during the 20th century. *Am J Clin Nutr. 93*(5), 950-962. https://doi.org/10.3945/ajcn.110.006643

Elbandy, M. (2022). Anti-inflammatory effects of marine bioactive compounds and their potential as functional food ingredients in the prevention and treatment of neuroinflammatory disorders. *Molecules 28*(1), 2. https://doi.org/10.3390/molecules28010002

Gómez Candela, C., Bermejo López, L. M. & Loria Kohen, V. (2011). Importance of a balanced omega 6/omega 3 ratio for the maintenance of health: nutritional recommendations. *Nutr Hosp 26*(2): 323-329. https://doi.org/10.1590/S0212-16112011000200013

Gow, R. V., & Hibbeln, J. R. (2014). Omega-3 fatty acid and nutrient deficits in adverse neurodevelopment and childhood behaviors. *Child Adolesc Psychiatr Clin NA*, *23*(3), 555-590. https://doi.org/10.1016/j.chc.2014.02.002

Hallahan, B., Ryan, T., Hibbeln, J. R., Murray, I. T., Glynn, S., Ramsden, C. E., SanGiovanni, J. P., & Davis, J. M. (2016). Efficacy of omega-3 highly unsaturated fatty acids in the treatment of depression. *Br J Psychiatry, 209*(3), 192-201. https://doi.org/10.1192/bjp.bp.114.160242

Hibbeln, J. R. and R. V. Gow (2014). "The potential for military diets to reduce depression, suicide, and impulsive aggression: a review of current evidence for omega-3 and omega-6 fatty acids." *Mil Med, 179*(11 Suppl), 117-128. https://doi.org/10.7205/MILMED-D-14-00153. PMID: 25373095

Jang, H. and K. Park (2020). "Omega-3 and omega-6 polyunsaturated fatty acids and metabolic syndrome: A systematic review and meta-analysis." *Clin Nutr, 39*(3), 765-773. https://doi.org/10.1016/j.clnu.2019.03.032

Lewis, M. D., Hibbeln, J. R., Johnson, J. E., Lin, Y. H., Hyun, D. Y. & Loewke, J. D. (2011). Suicide deaths of active-duty US military and omega-3 fatty-acid status: a case-control comparison. *J Clin Psychiatry, 72*(12):1585-1590. https://doi.org/10.4088/JCP.11m06879

Ramsden, C. E., Zamora, D., Leelarthaepin, B., Majchrzak-Hong, S. F., Faurot, K. R., Suchindran, C. M., Ringel, A., Davis, J. M. & Hibbeln, J. R. (2013). Use of dietary linoleic acid for secondary prevention of coronary heart disease and death: evaluation of recovered data from the Sydney Diet Heart Study and updated meta-analysis. *BMJ, 346:*e8707. https://doi.org/10.1136/bmj.e8707

Román, G. C., Jackson, R. E., Gadhia, R., Román, A. N. & Reis, J. (2019). Mediterranean diet: The role of long-chain ω-3 fatty acids in fish; polyphenols in fruits, vegetables, cereals, coffee, tea, cacao and wine; probiotics and vitamins in prevention of stroke, age-related cognitive decline, and Alzheimer disease. *Rev Neurol, 175*(10): 724-741. https://doi.org/10.1016/j.neurol.2019.08.005

Rousseau, G. (2021). Microbiota, a new playground for the omega-3 polyunsaturated fatty acids in cardiovascular diseases. *Mar Drugs 19*(2), 54. https://doi.org/10.3390/md19020054

Selmin, O. I., Papoutsis, A. J., Hazan, S., Smith, C., Greenfield, N., Donovan, M. G., Wren, S. N., Doetschman, T.C., Snider, J. M., Snider, A. J., Chow, S. H. & Romagnolo, D. F. (2021). *n*-6 High Fat Diet Induces Gut Microbiome Dysbiosis and Colonic Inflammation. *Int J Mol Sci, 22*(13), 6919. https://doi.org/10.3390/ijms22136919

Simopoulos, A. P. (2002). The importance of the ratio of omega-6/omega-3 essential fatty acids. *Biomed Pharmacother, 56*(8), 365-379. https://doi.org/10.1016/s0753-3322(02)00253-6

Simopoulos, A. P. (2006). "Evolutionary aspects of diet, the omega-6/omega-3 ratio and genetic variation: nutritional implications for chronic diseases." Biomed Pharmacother **60**(9), 502-507. https://doi.org/10.1016/j.biopha.2006.07.080

Simopoulos, A. P. (2008). The importance of the omega-6/omega-3 fatty acid ratio in cardiovascular disease and other chronic diseases. *Exp Biol Med, 233*(6), 674-688. https://doi.org/10.3181/0711-MR-311

Simopoulos, A. P. (2010). Genetic variants in the metabolism of omega-6 and omega-3 fatty acids: their role in the determination of nutritional requirements and chronic disease risk. *Exp Biol Med, 235*(7), 785-795. https://doi.org/10.1258/ebm.2010.009298

Simopoulos, A. P. (2016). An increase in the omega-6/omega-3 fatty acid ratio increases the risk for obesity. *Nutrients 8*(3), 128. https://doi.org/10.3390/nu8030128

Van Name, M. A., Savoye, M., Chick, J. M., Galuppo, B. T., Feldstein, A.E., Pierpont, B., Johnson, C., Shabanova, V., Ekong, U., Valentino, P. L., Kim, G., Caprio, S. & Santoro, N. (2020). A low ω-6 to ω-3 PUFA ratio (n-6:n-3 PUFA) diet to treat fatty liver disease in obese youth. *J Nutr, 150*(9):2314-2321. https://doi.org/10.1093/jn/nxaa183

Supplementary Table F References. Food Additives. Criteria to escape Tier III

Less than 2:1 ratio of kilocalories to milligrams of sodium

1. (Rust and Ekmekcioglu 2017)
2. (Fodor, Whitmore et al. 1999)
3. (Aliasgharzadeh, Tabrizi et al. 2022)
4. (Jafarnejad, Mirzaei et al. 2020)
5. (He, Li et al. 2013)
6. (He and MacGregor 2004)
7. (Neal, Wu et al. 2021)
8. (Fulgoni, Agarwal et al. 2014)
9. Action on salt consumer group report: https://www.actiononsalt.org.uk/media/action-on-salt/awareness/shake-the-salt-habit2022/Roundtable-Report_Accelerating-Salt-Reduction-in-the-UK.pdf
10. World Health Organization (WHO) online article\: https://www.who.int/europe/news/item/20-12-2018-european-salt-action-network-restates-its-support-for-who-goal-of-reducing-salt-intake-to-5-g-per-day-or-less

11. Johns Hopkins Bloomberg School of Public Health: https://globalhypertensionathopkins.org/courses/sodium

References

Action on Salt. (2022). Accelerating salt reduction in the UK. https://www.actiononsalt.org.uk/media/action-on-salt/awareness/shake-the-salt-habit2022/Roundtable-Report_Accelerating-Salt-Reduction-in-the-UK.pdf

Aliasgharzadeh, S., Tabrizi, J. S., Nikniaz, L., Ebrahimi-Mameghani, M. & Lotfi Yagin, N. (2022). Effect of salt reduction interventions in lowering blood pressure: a comprehensive systematic review and meta-analysis of controlled clinical trials. *PLoS One, 17*(12), e0277929. https://doi.org/10.1371/journal.pone.0277929

Fodor, J. G., Witmore, B., Leenen, F. & Larochelle, P. (1999). Lifestyle modifications to prevent and control hypertension. 5. Recommendations on dietary salt. Canadian Hypertension Society, Canadian Coalition for High Blood Pressure Prevention and Control, Laboratory Centre for Disease Control at Health Canada, Heart and Stroke Foundation of Canada. *CMAJ 160(*9 Suppl), S29-34.

Fulgoni, V. L., Agarwal, S., Spence, S. & Samuel, P. (2014). Sodium intake in US ethnic subgroups and potential impact of a new sodium reduction technology: NHANES Dietary Modeling. *Nutr J, 13*(1), 120. https://doi.org/10.1186/1475-2891-13-120

Global Hypertension at Hopkins. Global sodium reduction strategies. https://globalhypertensionathopkins.org/courses/sodium

He, F. J., Li, J. & MacGregor, G. A. (2013). Effect of longer-term modest salt reduction on blood pressure." *Cochrane Database Syst Rev, Apr 30*(4), CD004937. https://doi.org/10.1002/14651858.CD004937.pub2

Jafarnejad, S., Mirzaei, H., Clark, C. C. T., Taghizadeh, M. & Ebrahimzadeh, A. (2020). The hypotensive effect of salt substitutes in stage 2 hypertension: a systematic review and meta-analysis. *BMC Cardiovasc Disord, 20*(1), 98. https://doi.org/10.1186/s12872-020-01347-x

Neal, B., Wu, Y., Feng, X., Zhang, R., Zhang, Y., Shi, J., Zhang, J., Tian, M., Huang, L., Li, Z., Yu, Y., Zhao, Y., Zhou, B., Sun, J., Liu, Y., Yin, X., Hao, Z., Yu, J., Li, K. C., Zhang, X., Duan, P., Wang, F., Ma, B., Shi, W., Di Tanna, G. L., Stepien, S., Shan, S., Pearson, S. A., Li, N., Yan, L. L., Labarthe, D. & Elliott, P. (2021). Effect of salt substitution on cardiovascular events and death. *N Engl J Med,* 385(12), 1067-1077. https://doi.org/10.1056/NEJMoa2105675

Rust, P. & Ekmekcioglu, C. (2017). Impact of salt intake on the pathogenesis and treatment of Hypertension. *Adv Exp Med Biol, 956,* 61-84. https://doi.org/10.1007/5584_2016_147

World Health Organization. (2018). European Salt Action Network restates its support for WHO goal of reducing salt intake to 5 g per day or less. https://www.who.int/europe/news/item/20-12-2018-european-salt-action-network-restates-its-support-for-who-goal-of-reducing-salt-intake-to-5-g-per-day-or-less

Supplementary Table F References. Food Additives. Criteria to escape Tier III

Criterion: Trans fats

1. (Pipoyan, Stepanyan et al. 2021)
2. (Wallis, Bengtsson et al. 2022)
3. (Micha and Mozaffarian 2008)
4. (Micha and Mozaffarian 2008)
5. (Amico, Wootan et al. 2021)
6. (Hyseni, Bromley et al. 2017)
7. (Vinikoor, Millikan et al. 2010)
8. (de Souza, Mente et al. 2015)
9. (Oteng, Loregger et al. 2019)
10. (Oteng and Kersten 2020)

Amico, A., Wootan, M. G., Jacobson, M. F., Leung, C. & Willett, W. C. (2021). The demise of artificial trans fat: a history of a public health achievement. *Milbank Q, 99*(3), 746-770. https://doi.org/10.1111/1468-0009.12515

de Souza, R. J., Mente, A., Maroleanu, A., Cozma, A. I., Ha, V., Kishibe, T., Uleryk, E., Budylowski, P., Schünemann, H., Beyene, J. & Anand S. S. (2015). Intake of saturated and trans unsaturated fatty acids and risk of all-cause mortality, cardiovascular disease, and type 2 diabetes: systematic review and meta-analysis of observational studies. *BMJ, 351*, h3978. https://doi.org/10.1136/bmj.h3978

Hyseni, L., Bromley, H., Kypridemos, C., O'Flaherty, M., Lloyd-Williams, F., Guzman-Castillo, M., Pearson-Stuttard, J. & Capewell S. (2017). Systematic review of dietary trans-fat reduction interventions. *Bull World Health Organ, 95*(12), 821-830G. https://doi.org/10.2471/BLT.16.189795

Micha, R. & Mozaffarian, D. (2008). Trans fatty acids: effects on cardiometabolic health and implications for policy. *Prostaglandins Leukot Essent Fatty Acids 79*(3-5), 147-152. https://doi.org/10.1016/j.plefa.2008.09.008

Oteng, A. B. & Kersten, S. (2020). Mechanisms of action of trans fatty acids. *Adv Nutr 11*(3), 697-708. https://doi.org/10.1093/advances/nmz125

Oteng, A. B., Loregger, A., van Weeghel, M., Zelcer, N. & Kersten, S. (2019). Industrial trans fatty acids stimulate SREBP2-mediated cholesterogenesis and promote non-alcoholic fatty liver disease. *Mol Nutr Food Res, 63*(19), e1900385. https://doi.org/10.1002/mnfr.201900385

Pipoyan, D., Stepanyan, S., Stepanyan, S., Beglaryan, M., Costantini, L., Molinari, R. & Merendino, N. (2021). The effect of trans fatty acids on human health: regulation and consumption patterns. *Foods, 10*(10):2452. https://doi.org/10.3390/foods10102452

Vinikoor, L. C., Millikan, R, C., Satia, J. A., Schroeder, J. C., Martin, C. F., Ibrahim, J. G. & Sandler, R. S. (2010). Trans-fatty acid consumption and its association with distal colorectal cancer in the North Carolina Colon Cancer Study II. *Cancer Causes Control, 21*(1), 171-180. https://doi.org/10.1007/s10552-009-9447-3

Wallis, J. G., Bengtsson, J. D. & Browse, J. (2022). Molecular approaches reduce saturates and eliminate trans fats in food oils. *Front Plant Sci, 13,* 908608. https://doi.org/10.3389/fpls.2022.908608

Supplementary Table G. Criteria to achieve Tier II

Criterion	Comment	Tier II C	Tier II B	Tier II A
Escapes Tier III by scientific evidence of absence of detrimental health effects		●	●	●
No oil that has more than 40% omega-6 fatty acids (sunflower, cottonseed, soybean, canola/rapeseed, or corn oil)			●	●
At least 950 IU (= 285 mcg of retinol) of vitamin A per serving	50% AR adult males			●
At least 45 mg of vitamin C per serving	50% AR adult males			●
At least 400 IU (= 10 mcg) of vitamin D per serving	50% RDA adults			●
At least 9.69 IU (= 6.5 mg of α-tocopherol) of vitamin E per serving	50% AI adult males			●
At least 200 mg of elemental calcium per serving (equivalent to 0.5 g of calcium carbonate or 2 g of calcium citrate/calcium citrate malate)	50% RDA adults		at least one	●
At least 200 µg of folate per serving	50% RDA adults			●
At least 4 mg of elemental iron per serving	50% RDA children			●
At least 175 mg of magnesium per serving	50% AI adult males			●
At least 5.5 mg of zinc per serving	50% RDA adult males			●
At least 250 mg of combined EPA/DHA per serving	50% RDA adults			●
At least 1 g of ALA per serving	50% RDA adults			●
Dietary fiber ratio (to carbohydrate) >1:10				●

Supplementary Tables References

Supplementary Table G. Criteria to achieve Tier II

No oil that has more than 40% omega-6 fatty acids
(sunflower, cottonseed, soybean, canola/rapeseed, or corn oil)

1.(Simopoulos 2016)
2. (Hibbeln, Nieminen et al. 2006)
3. (Patterson, Wall et al. 2012)
4. (Ascherio and Willett 1997)
5. (Tarrago-Trani, Phillips et al. 2006)
6. (Misra, Singhal et al. 2010)
7. (Wendell, Baffi et al. 2014)
8. (Fernandes 1994)
9. (Berger, Smesny et al. 2017)
10. (Loef and Walach 2013)
11. (Lauretti and Praticò 2017)
12. (Golomb and Bui 2015)
13. (Golomb, Evans et al. 2012)
14. (Alvheim, Malde et al. 2012)
15. (Deol, Evans et al. 2015)
16. (Deol, Evans et al. 2015)
17. (Rudolph, Young et al. 2017)

18. (Simopoulos 2016)
19. (DiNicolantonio and O'Keefe 2018)
20. (Patterson, Wall et al. 2012)

References:

Alvheim, A. R., Malde, M. K., Osei-Hyiaman, D., Lin, Y. H., Pawlosky, R. J., Madsen, L., Kristiansen, K., Frøyland, L. & Hibbeln, J. R. (2012). Dietary linoleic acid elevates endogenous 2-AG and anandamide and induces obesity. *Obesity, 20*(10), 1984-1994. https://doi.org/10.1038/oby.2012.38

Ascherio, A. and Willett, W. C. (1997). Health effects of trans fatty acids. *Am J Clin Nutr, 66*(4 Suppl), 1006s-1010s. https://doi.org/10.1093/ajcn/66.4.1006S

Berger, M. E., Smesny, S., Kim, S. W., Davey, C. G., Rice, S., Sarnyai, Z., Schlögelhofer, M., Schäfer, M. R., Berk, M., McGorry, P. D., Amminger, G. P. (2017) Omega-6 to omega-3 polyunsaturated fatty acid ratio and subsequent mood disorders in young people with at-risk mental states: a 7-year longitudinal study. *Transl Psychiatry, 7*(8):e1220. https://doi.org/10.1038/tp.2017.190

Deol P, Evans JR, Dhahbi J, Chellappa K, Han DS, Spindler S, Sladek FM. Soybean oil Is more obesogenic and diabetogenic than coconut oil and fructose in mouse: potential role for the liver. *PLoS One, 10*(7):e0132672. https://doi.org/10.1371/journal.pone.0132672
DiNicolantonio, J. J. & O'Keefe, J. H. (2018). Omega-6 vegetable oils as a driver of coronary heart disease: the oxidized linoleic acid hypothesis. *Open Heart 5*(2), e000898. https://doi.org/10.1136/openhrt-2018-000898

Fernandes, G. (1994). Dietary lipids and risk of autoimmune disease. *Clin Immunol Immunopathol, 72*(2), 193-197. https://doi.org/10.1006/clin.1994.1129

Golomb, B. A. & Bui, A. K. (2015). A fat to forget: trans fat consumption and memory. *PLoS One 10*(6), e0128129. https://doi.org/10.1371/journal.pone.0128129

Golomb, B. A., Evans, M. A., White, H. L. & Dimsdale, J. E. (2012). Trans fat consumption and aggression. *PLoS One 7*(3), e32175. https://doi.org/10.1371/journal.pone.0032175

Hibbeln, J. R., Nieminen, L. R., Blasbalg, T. L., Riggs, J. A. & Lands, W.E. (2006). Healthy intakes of n-3 and n-6 fatty acids: estimations considering worldwide diversity. *Am J Clin Nutr, 83*(6 Suppl), 1483S-1493S. https://doi.org/10.1093/ajcn/83.6.1483S

Lauretti, E. & Praticò, D. (2017). Effect of canola oil consumption on memory, synapse and neuropathology in the triple transgenic mouse model of Alzheimer's disease. *Sci Rep 7*(1), 17134. https://doi.org/10.1038/s41598-017-17373-3

Loef, M. & Walach, H. (2013). The omega-6/omega-3 ratio and dementia or cognitive decline: a systematic review on human studies and biological evidence. *J Nutr Gerontol Geriatr 32*(1), 1-23. https://doi.org/10.1080/21551197.2012.752335

Misra, A., Singhal, N. & Khurana, L. (2010). Obesity, the metabolic syndrome, and type 2 diabetes in developing countries: role of dietary fats and oils. *J Am Coll Nutr 29*(3 Suppl): 289s-301s. https://doi.org/10.1080/07315724.2010.10719844

Patterson, E., Wall, R., Fitzgerald, Ross, R. P. & Stanton, C. (2012). Health implications of high dietary omega-6 polyunsaturated Fatty acids. *J Nutr Metab, 2012:* 539426. https://doi.org/10.1155/2012/539426

Rudolph, M. C., Young, B. E., Lemas, D. J., Palmer, C. E., Hernandez, T. L., Barbour, L. A., Friedman, J. E., Krebs, N. F. & MacLean, P. S. (2017). Early infant adipose deposition is positively associated with the n-6 to n-3 fatty acid ratio in human milk independent of maternal BMI. *Int J Obes 41*(4), 510-517. https://doi.org/10.1038/ijo.2016.211

Simopoulos, A. P. (2016). An increase in the omega-6/omega-3 fatty acid ratio increases the risk for obesity. *Nutrients 8*(3), 128. https://doi.org/10.3390/nu8030128

Tarrago-Trani, M. T., Phillips, K. M., Lemar, L. E. & Holden, J. M. (2006). New and existing oils and fats used in products with reduced trans-fatty acid content. *J Am Diet Assoc 106*(6): 867-880. https://doi.org/10.1016/j.jada.2006.03.010

Wendell, S. G., Baffi, C. & Holguin, F. (2014). Fatty acids, inflammation, and asthma. *J Allergy Clin Immunol 133*(5): 1255-1264. https://doi.org/10.1016/j.jaci.2013.12.1087

Supplementary Table H1. Health criteria to achieve Tier I

Criterion	Tier I B	Tier I A
At least 0.75 mg of vitamin B6 (pyridoxine) per serving	●	●
At least 2 µg of vitamin B12 (cobalamin) per serving	●	●
Information about the presence/absence of "Big 8" allergens (milk, eggs, fish, crustacean shellfish, tree nuts, peanuts, wheat, and soybean)	●	●
Positive health effects supported explicitly by scientific evidence	●	●
The product has been chemically tested by an independent body		●
Only cold-pressed plant-based oils		●
At least 88 mg of tryptophan per serving		●

Supplementary Table H1 References. Health criteria to achieve Tier I

Refs 1-5: At least 0.75 mg of vitamin B6 (pyridoxine) per serving

1. (Ueland, McCann et al. 2017)
2. (Selhub, Byun et al. 2013)
3. (Wan, Zheng et al. 2022)
4. (Stach, Stach et al. 2021)
5. National Institutes of Health, US Department of Health & Human Services online article: https://ods.od.nih.gov/factsheets/VitaminB6-HealthProfessional

Refs 6-10: At least 2 µg of vitamin B12 (cobalamin) per serving

6. National Institutes of Health, US Department of Health & Human Services online article: https://ods.od.nih.gov/factsheets/VitaminB12-HealthProfessional/
7. (Stach, Stach et al. 2021)
8. (Rahman and Baumgartner 2019)
9. (Hossain, Amarasena et al. 2022)
10. (Mikkelsen and Apostolopoulos 2018)

Refs 11-15: Only cold-pressed plant-based oils

11. (Durazzo, Fawzy Ramadan et al. 2021)
12. (Fratianni, d'Acierno et al. 2021)

13. (De Santis, Cariello et al. 2019)
14. (Romani, Ieri et al. 2019)
15. (Prescha, Grajzer et al. 2014)

Refs 16-20: At least 88 mg of tryptophan per serving

16. (Kikuchi, Tanabe et al. 2021)
17. (Kałużna-Czaplińska, Gątarek et al. 2019)
18. (Gibson 2018)
19. (Dell'Osso, Carmassi et al. 2016)
20. (Agus, Planchais et al. 2018)

References:

Agus, A., Planchais, J. & Sokol, H. (2018). Gut microbiota regulation of tryptophan metabolism in health and disease. *Cell Host Microbe 23*(6): 716-724. https://doi.org/10.1016/j.chom.2018.05.003

De Santis, S., Cariello, M., Piccinin, E., Sabbà, C. & Moschetta, A. (2019). Extra virgin olive oil: lesson from nutrigenomics. *Nutrients 11*(9), 2085. https://doi.org/10.3390/nu11092085

Dell'Osso, L., Carmassi, C., Mucci, F. & Marazziti, D. (2016). Depression, serotonin and tryptophan. *Curr Pharm Des, 22*(8):949-954. https://doi.org/10.2174/1381612822666151214104826

Durazzo, A., Fawzy Ramadan, M. & Lucarini, M. (2021). Editorial: Cold pressed oils: a green source of specialty oils. *Front Nutr, 8*, 836651. https://doi.org/10.3389/fnut.2021.836651

Fratianni, F., d'Acierno, A., Ombra, M. N., Amato, G., De Feo, V., Ayala-Zavala, J. F., Coppola, R. & Nazzaro, F. (2021). Fatty acid composition, antioxidant, and *in vitro* anti-inflammatory activity of five cold-pressed *Prunus* seed oils, and their anti-biofilm effect against pathogenic bacteria. *Front Nutr, 8*, 775751. https://doi.org/10.3389/fnut.2021.775751

Gibson, E. L. (2018). Tryptophan supplementation and serotonin function: genetic variations in behavioural effects. *Proc Nutr Soc 77*(2), 174-188. https://doi.org/10.1017/S0029665117004451

Hossain, K. S., Amarasena, S. & Mayengbam, S. (2022). B vitamins and their roles in gut health. *Microorganisms 10*(6), 1168. https://doi.org/10.3390/microorganisms10061168

Kałużna-Czaplińska, J., Gątarek, P., Chirumbolo, S., Chartrand, M. S. & Bjørklund G. (2019). How important is tryptophan in human health? *Crit Rev Food Sci Nutr, 59*(1), 72-88. https://doi.org/10.1080/10408398.2017.1357534

Kikuchi, A. M., Tanabe, A. & Iwahori, Y. (2021). A systematic review of the effect of L-tryptophan supplementation on mood and emotional functioning. *J Diet Suppl 18*(3), 316-333. https://doi.org/10.1080/19390211.2020.1746725

Mikkelsen, K. & Apostolopoulos, V. (2018). "B Vitamins and Ageing." Subcell Biochem **90**: 451-470. https://doi.org/10.1007/978-981-13-2835-0_15

National Institutes of Health. Vitamin B6. https://ods.od.nih.gov/factsheets/VitaminB6-HealthProfessional/

National Institutes of Health. Vitamin B12. https://ods.od.nih.gov/factsheets/VitaminB12-HealthProfessional/

Prescha, A., Grajzer, M., Dedyk, M. & Grajeta, H. (2014). The antioxidant activity and oxidative stability of cold-pressed oils. *Journal of the American Oil Chemists' Society 91*(8), 1291-1301. https://doi.org/10.1007/s11746-014-2479-1

Rahman, S. & Baumgartner, M. (2019). B vitamins: small molecules, big effects. J Inherit Metab Dis **42**(4): 579-580. https:/doi.org/10.1002/jimd.12127

Romani, A., et al. (2019). Health Effects of Phenolic Compounds Found in Extra-Virgin Olive Oil, By-Products, and Leaf of Olea europaea L. *Nutrients 11*(8), 1776. https://doi.org/10.3390/nu11081776

Selhub, J., Byun, A., Liu, Z., Mason, J. B., Bronson, R. T., Crott, J. W. (2013). Dietary vitamin B6 intake modulates colonic inflammation in the IL10-/- model of inflammatory bowel disease. *J Nutr Biochem, 24*(12):2138-2143. https://doi.org/10.1016/j.jnutbio.2013.08.005

Stach, K., Stach, W. & Augoff, K. (2021). Vitamin B6 in Health and Disease. *Nutrients 13*(9), 3229. https://doi.org/10.3390/nu13093229

Ueland, P. M., McCann, A., Middtun, Ø. & Ulvik, A. (2017). Inflammation, vitamin B6 and related pathways. *Mol Aspects Med 53,* 10-27. https://doi.org/10.1016/j.mam.2016.08.001

Wan, Z., Zheng, J., Zhu, Z., Sang, L., Zhu, J., Luo, S., Zhao, Y., Wang, R., Zhang, Y., Hao, K., Chen, L., Du, J., Kan, J. & He, H. (2022). Intermediate role of gut microbiota in vitamin B nutrition and its influences on human health. *Front Nutr, 9,* 1031502. https://doi.org/10.3389/fnut.2022.1031502

Supplementary Table H2. Environmental, sustainability and market criteria to achieve Tier I

<table>
<tr><th>Criterion</th><th>Tier II B</th><th>Tier II A</th></tr>
<tr><td>Products sourced from humanely raised animals
(continuous pasture access, independently verified)</td><td>●</td><td>●</td></tr>
<tr><td>Certified organic</td><td rowspan="4">as many as possible</td><td rowspan="8">as many as possible</td></tr>
<tr><td>Non-GMO</td></tr>
<tr><td>Traceable sourcing</td></tr>
<tr><td>Recyclable or compostable packaging in line with current KDD efforts</td></tr>
<tr><td>Sets a standard for the entire industry</td><td></td></tr>
<tr><td>Transform the portfolio of products sold in KDD retail outlets</td><td></td></tr>
<tr><td>Marketing values that align with current best practices</td><td></td></tr>
<tr><td>Environmentally sourced
(small production, processing, distribution, carbon footprint, etc.)</td><td></td></tr>
<tr><td>Meets UN Sustainable Development Goals (SDGs)
No. 3 (“Ensure healthy lives and promote well-being for all at all ages”) and
No. 12 (“Ensure sustainable consumption and production patterns”)</td><td></td></tr>
</table>

Supplementary Table H2 References. Environmental, sustainability and market criteria to achieve Tier I

Refs 1-4 Humane Farming

1. FACT online article about humane farming: https://www.foodanimalconcernstrust.org/nutritional-benefits
2. The Humane Farming Association: https://www.hfa.org
3. Compassion in world farming: https://www.ciwf.org.uk/research/solutions-for-humane-and-sustainable-agriculture
4. Humane Society International: https://www.hsi.org/issues/factory-farming

Refs 5-8 Organic Foods

5. (Mie, Andersen et al. 2017)
6. (Hurtado-Barroso, Tresserra-Rimbau et al. 2019)
7. (Brantsæter, Ydersbond et al. 2017)
8. Soil Association online article: The Benefits of Organic Farming: file:///Users/rachelvictoria/Downloads/the-benefits-of-organic-farming-april-2018%20(1).pdf and https://www.soilassociation.org/policy-reports

Refs 9-12: Non-GMO

9. (Salt 2023)
10. (Buchholzer and Frommer 2023)
11. The Non-GMO Project: https://www.nongmoproject.org/gmo-facts/what-is-gmo
12. Center for Food Safety: https://www.centerforfoodsafety.org/press-releases/3766/are-gmos-safe-no-consensus-in-the-science-scientists-say-in-peer-reviewed-statement

Refs 13-16: Recyclable or compostable packaging

13. Online article: https://wrap.org.uk/resources/guide/compostable-plastic-packaging-guidance
14. European bioplastics: https://www.european-bioplastics.org/faq-items/what-are-the-required-circumstances-for-a-compostable-product-to-compost
15. Food Standards Agency: https://www.food.gov.uk/sites/default/files/media/document/bio-based-materials-for-use-in-food-contact-applications_0.pdf
16. European Economic and Social Committee: https://www.eesc.europa.eu/sites/default/files/files/qe-03-20-534-en-n.pdf

Refs 17-19: Environmentally sourced

17. (Karwacka, Ciurzyńska et al. 2020)
18. (Xu, Sun et al. 2015)
19. (Russell 2014)

Refs 20:22: Meets UN Sustainable Development Goals (SDGs)

20. (Palmer 2015)
21. (Bexell and Jönsson 2017)
22. United nations: https://www.un.org/sustainabledevelopment/sustainable-development-goals

References:

Bexell, M. & Jönsson, K. (2017). Responsibility and the United Nations' Sustainable Development Goals." *Forum for Development Studies 44*(1), 13-29. https://doi.org/10.1080/08039410.2016.1252424

Brantsæter, A. L., Ydersbond, T. A., Hoppin, J. A., Haugen, M. & Meltzer, H. M. (2017). Organic food in the diet: exposure and health implications. *Ann Rev Public Health, 38*, 295-313. https://doi.org/10.1146/annurev-publhealth-031816-044437

Buchholzer, M. & Frommer, W. B. (2023). An increasing number of countries regulate genome editing in crops. *New Phytologist 237*(1), 12-15. https://doi.org/10.1111/nph.18333

Center for Food Safety. Are GMOs safe? No consensus in the science, scientists say in peer-reviewed statement https://www.centerforfoodsafety.org/press-releases/3766/are-gmos-safe-no-consensus-in-the-science-scientists-say-in-peer-reviewed-statement

Food and Animal Concerns Trust. Nutritional benefits of humane farming. https://www.foodanimalconcernstrust.org/nutritional-benefits

Hurtado-Barroso, S., Tresserra-Rimbau, A., Vallverdú-Queralt, A. & Lamuela-Raventós, R. M. (2019). Organic food and the impact on human health. *Crit Rev Food Sci Nutr 59(*4), 704-714. https://doi.org/10.1080/10408398.2017.1394815

Karwacka, M.; Ciurzyńska, A.; Lenart, A. & Janowicz, M. (2020). Sustainable Development in the Agri-Food Sector in Terms of the Carbon Footprint: A Review. *Sustainability 12*, 6463. https://doi.org/10.3390/su12166463

Mie, A., Andersen, H.R., Gunnarsson, S., Kahl, J., Kesse-Guyot, E., Rembiałkowska, E., Quaglio, G. & Grandjean P. (2017). Human health implications of organic food and organic agriculture: a comprehensive review. *Environ Health, 16*(1), 111. https://doi.org/10.1186/s12940-017-0315-4

Non-GMO Project. What is a GMO? https://www.nongmoproject.org/gmo-facts/what-is-gmo/

Palmer, E. (2015). INTRODUCTION: The Sustainable Development Goals Forum. *Journal of Global Ethics 11*(1), 3-9. https://philarchive.org/archive/PALTSD-2

Russell, D. A. M. (2014). Sustainable (food) packaging – an overview. *Food Additives & Contaminants: Part A 31*(3), 396-401. https://doi.org/10.1080/19440049.2013.856521

Salt, D. E. (2023). GMO or non-GMO? That is the question. *New Phytologist 237*(1): 7-8. https://doi.org/10.1111/nph.18399

Wrap. Compostable plastic packaging guidance. https://wrap.org.uk/resources/guide/compostable-plastic-packaging-guidance

Xu, Z., Sun, D. W., Zeng, X. A., Liu, D. & Pu, H. (2015). Research developments in methods to reduce the carbon footprint of the food System: a review." *Crit Rev Food Sci Nutr, 55*(9): 1270-1286. https://doi.com/10.1080/10408398.2013.821593
